# Expanding first-line options for depression: Protocol of a pragmatic comparative effectiveness trial of yoga vs. behavioral activation (the COMPARE study)

**DOI:** 10.1371/journal.pone.0315506

**Published:** 2025-01-06

**Authors:** Louisa G. Sylvia, Andrew M. Busch, Dustin J. Rabideau, Alexandra Gold, Suzanne C. Danhauer, Heather T. Schatten, Douglas Katz, Lauren M. Weinstock, Jennifer Dahne, Sabine P. Schmid, Zainab Soetan, Roberta Tovey, Kedie Pintro, Adrienne Kvaka, Antonietta Alvarez Hernandez, Ingrid Hsu, Alba Melendez, Melissa Adkins-Hempel, Angela Grubb, Odalys Lozado, Atefeh Alavi Fili, Giuliana Chau, Lisa A. Uebelacker

**Affiliations:** 1 Massachusetts General Hospital, Boston, Massachusetts, United States of America; 2 Harvard Medical School, Boston, Massachusetts, United States of America; 3 Hennepin Healthcare, Minneapolis, Minnesota, United States of America; 4 University of Minnesota Twin Cities, Minneapolis, Minnesota, United States of America; 5 Atrium Health Wake Forest Baptist, Wake Forest, North Carolina, United States of America; 6 Warren Alpert Medical School of Brown University, Providence, Rhode Island, United States of America; 7 Medical University of South Carolina, Charleston, South Carolina, United States of America; 8 Butler Hospital, Providence, Rhode Island, United States of America; Public Library of Science, UNITED STATES OF AMERICA

## Abstract

Depression is a prevalent mental health condition in the United States and a significant cause of morbidity and mortality. The treatment guidelines for depression recommends either psychotherapy, such as behavioral activation (BA), or a second-generation antidepressant as a first-line treatment for adult patients with depression. However, many individuals with depression do not experience improvement from first-line treatments or choose not to engage in them due to stigma, cost, difficulty with access, and/or side effects. As such we need new treatments for depression and yoga is especially promising given recent data on its efficacy for depression. This study seeks to compare a first-line treatment for depression, or BA, versus yoga to examine whether yoga does as well as BA at improving depressive symptoms and secondary outcomes. We will also examine improvements in depressive symptoms, and secondary outcomes, by specific sub-populations to determine who might do better in which treatment (i.e., BA or yoga). Given that this is the first non-inferior, comparative effectiveness study of yoga, this paper explains the study design, the rationale for the study design, as well as lessons learned in conducting the study.

## Introduction

Depression is a prevalent mental health condition in the United States [1] and a significant cause of morbidity and mortality. Depressive disorders are also the second leading cause of disease burden globally, representing 6.2% of this global burden, and were anticipated to be the leading cause of global disease burden by 2030 [2]. Depression is also associated with high risk of mortality from medical conditions (i.e., cardiovascular disease, ischemic disease [3]) and suicide-related behaviors [4]. Moreover, individuals who do not meet full criteria for a major depressive disorder, but endorse elevated symptoms of depression, still experience many of the negative consequences of depression, including detrimental effects on quality of life [5], financial strain [6], heightened risk for developing MDD [7], and elevated mortality risk [8]. Thus, there is a need for timely and cost-effective interventions that can be easily disseminated and prevent or alleviate the impact of depressive symptoms.

The American Psychological Association’s (APA) 2019 Clinical Practice Guideline recommends specific treatments for depression, reviews their benefits and risks, and evaluates the relevance of specific interventions across demographic populations and environments [9]. This guideline recommends either psychotherapy or a second-generation antidepressant as a first-line treatment for adult patients with depression [9]. Recommended psychotherapies include behavioral, cognitive, cognitive-behavioral, and mindfulness-based cognitive therapy, interpersonal psychotherapy, psychodynamic therapies, and supportive therapies [9].

Behavioral therapies are rooted in behavioral theory of depression [10, 11], which posits that depression is caused by lack of contact with response contingent positive reinforcement. This lack of contact can be caused by many interacting factors, including chronically low of availability of positive reinforcement, loss of a primary source of positive reinforcement (e.g., job loss, divorce), lack of the skill repertoire to access available positive reinforcers, and an environment that provides more support for depressive behaviors (i.e., escape or avoidance, or other behaviors not in line with long term values) than for non-depressed behaviors (i.e., approach behaviors that are in line with long term values) [11–13]. Behavioral activation (BA) is a type of behavioral therapy that is brief, present-focused, and aims to increase individuals’ access to and engagement with positive reinforcement in their environment [14, 15].

Early BA treatments focused only on increasing “pleasant events”, however later BA manuals expanded this target to behaviors that may not be pleasant in the moment but, are likely antidepressant in the long run (e.g., behaviors consistent with personal life values) [16, 17]. BA has strong research support for the treatment of depression, with large-scale randomized controlled trials demonstrating its efficacy [18, 19] and meta-analyses reflecting the superiority of BA relative to control treatments [20, 21]. BA is less complex than CBT and thus, appears to require less training and can be conducted with fidelity by interventionists with less expertise [19, 22, 23]. There is also no difference in outcomes for depression when delivering BA via telehealth (phone, video) versus in-person [24], thus increasing its accessibility. Further, there is evidence suggesting that therapists can be effectively trained in BA remotely with limited training time [25, 26]. In sum, BA is efficacious for depression, relatively easy to teach to therapists, and accessible via telehealth.

Despite the demonstrated efficacy of first-line treatments for depression such as BA or pharmacotherapy, these treatments may not be ideal for all individuals with depression. Many individuals with depression do not experience improvement from first-line treatments, desire treatments that are better aligned with their own values or belief systems, are unable to tolerate medication side effects [27], experience difficulties accessing or affording such treatments [28, 29], and experience stigma surrounding seeking mental health treatment. Therefore, many may not engage with current first-line treatments for depression [30]. As such, it is essential that we identify additional interventions that can lead to symptom relief and improved well-being for those with depressive symptoms.

Data suggest that up to 50% of individuals with a diagnosis of depression use complementary and integrative treatments [31] such as yoga or exercise. In particular, yoga is especially promising as a treatment for depression. Yoga interventions have shown efficacy across meta-analyses for improving depressive symptom severity among individuals with elevated depressive symptoms [27, 32–34]. Hatha yoga, which includes physical postures, attention to the breath, breathing exercises, and meditation, represents the most commonly practiced form of yoga in the United States [35]. Yoga practice may improve depressive symptoms by encouraging a non-judgmental orientation to the present-moment (i.e., mindfulness), thereby reducing the rumination and self-criticism that can contribute to depressive symptoms [36]. Yoga breath practices may contribute to reduced activation of the sympathetic nervous system and increased activation of the parasympathetic nervous system, thereby promoting self-regulation in response to stress [37]. There are several reasons why individuals may be interested in pursuing yoga in lieu of traditional treatments for depression. In addition to demonstrating effectiveness for depressive symptoms [27, 32–34], yoga avoids the stigma that is often associated with mental health treatment [38], is increasingly popular [39] and can improve physical health symptoms (e.g., chronic pain) that are often comorbid with depression [40]. Similar to BA, yoga can easily and effectively be administered via telehealth [41, 42]. There are numerous free, high quality asynchronous (pre-recorded) yoga resources and classes available to support the practice of yoga [43, 44], making the continued practice more sustainable. Thus, yoga has been demonstrated to be an efficacious, sustainable, cost effective, and accessible treatment for depression [45, 46].

Although data for the efficacy of yoga interventions for depression are promising, yoga interventions have yet to be demonstrated as non-inferior to a first-line treatment for depression [47]. Treatment guidelines from organizations such as the APA serve as an important resource which may be used to argue that particular treatments have sufficient evidence base and should be covered by insurance [48]. Thus, a non-inferiority clinical trial that supports the comparative benefit of a yoga intervention to a first-line treatment for depression that is covered by insurance, such as BA, could lead to updated guidelines recognizing yoga as a first-line treatment option for depression. Treatment guidelines for depression from organizations outside of the US (i.e., the National Institute of Clinical Excellence guidelines in the United Kingdom) [9, 49] recognize yoga as a first-line treatment option for depression.

This paper describes a pragmatic, multi-site, randomized non-inferiority trial comparing two interventions for depression among adults with mild to moderate depression symptoms funded by Patient-Centered Outcomes Research Institute (PCORI) [50]. Specifically, this study aims to: (1) evaluate the noninferiority of a synchronous yoga intervention (YI) to an established psychotherapy, namely behavioral activation (BA) for treating depression (both delivered via telehealth and offered for 12 weeks); and (2) examine participant-level characteristics that predict outcomes to ultimately guide matching participants to interventions (see Table 1). Given that this study is the first pragmatic, comparative effectiveness trial of a YI compared to a first-line treatment for depression delivered via telehealth, we present the study procedures to support replication of our findings, enhance interpretation of our results, and inform researchers for future clinical trials of YIs and other complementary and integrative interventions for depression.

**Table 1:** Study hypotheses.

*Hypotheses*	
**1a**	The YI will be non-inferior to BA on the primary outcome of depression symptoms at 24 weeks (i.e., 6 months).
**1b**	The YI will be non-inferior to BA on the secondary outcomes of overall well-being, anxiety, and sleep at 24 weeks (i.e., 6 months).
**2**	There will be greater effectiveness of the YI compared to BA among participants with a stronger preference for the YI, whereas there will be greater effectiveness of BA compared to the YI among participants with a stronger preference for BA.
**3**	There will be greater effectiveness of the YI compared to BA among participants with higher perceived credibility of the YI relative to BA, whereas there will be greater effectiveness of BA compared to the YI among participants with higher perceived credibility of BA relative to the YI.
**4**	There will be greater effectiveness of BA compared to the YI among participants with more severe depression at baseline.
**5**	There will be greater effectiveness of the YI compared to BA among participants with a higher degree of trust in their bodies.

## Study design

### Study design overview

Participants are randomly assigned in a parallel group study design to either a telehealth, synchronous group YI for individuals with depression [51] or to individual, clinician-delivered telehealth BA for depression [16]. Participant involvement lasts for 24 weeks and consists of a 12-week treatment period (i.e., either 1 weekly yoga class or 8 BA sessions over 12 weeks) and a 12-week post-treatment follow-up period. The primary outcome is depression symptom severity (assessed via the Patient Health Questionaire-9; PHQ-9) [52] at 24 weeks (i.e., 6 months). Secondary outcomes include overall well-being, anxiety symptoms, and sleep.

Participants (target N = 518) are recruited and randomized across four collaborating institutions: Massachusetts General Hospital (Boston, Massachusetts), Hennepin Healthcare Research Institute (Minneapolis, Minnesota), Butler Hospital (Providence, Rhode Island), and Wake Forest University Health Sciences (Winston-Salem, NC). The study has been approved by the Mass General Brigham Human Research Protection Program (IRB) on September 9^th^, 2022 (IRB; #2022P001701, NCT05546697). Recruitment period began on January 1^st^, 2023 and will end on May 1^st^, 2026.

### Participants

Participants are included based on the following criteria. They must be a) adults (≥18 years old) with b) depressive symptoms at study entry (PHQ-9≥10) who c) provide written informed consent, d) are able to read and understand English or Spanish, e) live in one of the four states in which therapists are licensed (NC, MA, MN, or RI), and f) have a healthcare provider they can contact if they need medical care (including a primary care doctor, clinic, or mental health clinician of some type). We exclude children and teens as there are important ways in which yoga needs to be modified to be acceptable to them. We do not exclude older adults as the yoga program will be gentle and low impact and thus, appropriate for them provided they meet other eligibility criteria. We require a healthcare provider to ensure that, if participants are at increased risk for suicide, there is a health care provider they can contact in addition to being able to call 988.

We exclude participants who: a) have had a bone fracture or joint surgery in the past 6 months, b) are unable to walk, c) have severe heart failure or lung disease, d) have had a healthcare provider tell them it is unsafe to exercise, e) are pregnant, f) are already engaged in study interventions (i.e., engaged in yoga practice or psychotherapy more than once in the past 4 weeks, or has an intake for psychotherapy scheduled for the next 4 weeks), or g) are severely depressed (i.e., PHQ-9>20), (h) have active suicidal thinking (i.e., PHQ-9 item 9 ≥1 and a positive response to CSSR-S screener items 2, 3, 4, 5, or 6) or i) are currently manic (Altman Self-Rating Mania Scale score ≥ 6) [53].

Criterion “a” through “e” are to ensure participant safety in yoga. The yoga intervention is gentle and therefore, accessible to people with chronic pain and other health conditions (e.g., obesity) [54]. We exclude pregnant women because we recommend that pregnant women enroll in classes specifically geared toward them and with teachers who are knowledgeable about pregnancy. We exclude individuals engaged in the study interventions to minimize treatment non-compliance or contamination. Further, many psychotherapy sessions include aspects of BA and some insurances will not pay for therapy more than once per week. Finally, we exclude individuals who may need more intensive treatment for their depression than the study interventions, which are appropriate for people with mild to moderate depression, but likely not ideal as a monotherapy for people with more complex or severe presentations. We do include people with bipolar depression as they cannot be reliably differentiated from those with unipolar depression, respond similarly to yoga and BA interventions as those with unipolar depression, and often choose to engage in yoga and BA interventions [55–64]. In sum, the proposed sample is broad and should be generalizable to many people with depression given we include people with comorbid conditions as well as those taking pharmacotherapy for depression.

### Recruitment procedures

Given that previous studies of YIs have tended to disproportionately enroll participants who identify as women and white, we have dedicated study funds and staff to support recruitment via multiple avenues to enhance the generalizability of study findings to other subpopulations. We advertise the study in English and Spanish and through culturally informed social media advertisements, messages sent via patient health portals associated with electronic health records, established partnerships with national and local organizations (e.g., the Depression and Bipolar Support Alliance), and at community health fairs and other community events. Finally, we also prioritize outreach via targeted advertisements to community-based primary care facilities. Overall, we develop advertising materials to include images of people with varying body sizes, of varying ages, and with varying gender, racial, and ethnic identities. We include images with both rural and urban backgrounds, and, in response to previous feedback from people with depression, images of people looking happy or content (rather than sad or down). Advertisements emphasize comparative effectiveness, without suggesting one intervention is superior to the other.

### Study procedures

**Fig 1:**
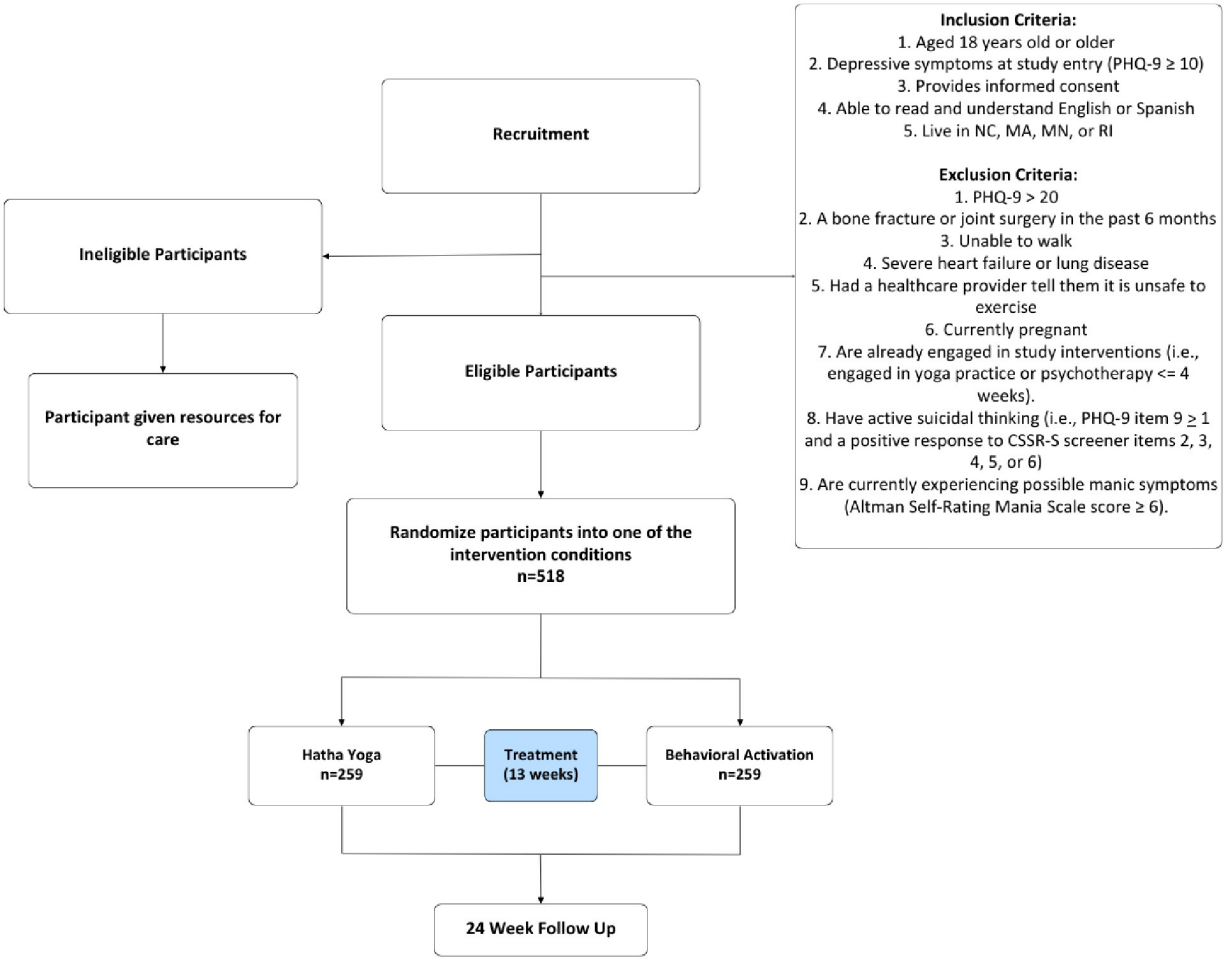
COMPARE study schema.

The primary mode of data collection occurs through a secure, electronic data capture system in REDCap [65]. Individuals interested in participating in the study click on a link to go to the study website which describes the study and includes contact information for study staff and a link to the REDCap pre-screening questionnaire. If participants are initially eligible via the pre-screening questionnaire, they are given a study information sheet which describes the study procedures, risks and benefits to study participation, and provides study team contact information. This information sheet was approved by the Mass General Brigham IRB to serve as the study’s informed written consent given that the study is considered low-risk and thus, does not require a signature for participation. After reviewing the information sheet on REDCap and indicating interest in participating, study staff contact participants for a “teach-back call.” This telephone call (or series of calls) is intended to ensure that participants have a comprehensive understanding of study procedures, risks, and benefits. Staff answer participants’ questions, check understanding of study procedures, and confirm that participants agree to randomization. Study staff collect key pieces of information: insurance information (to determine out-of-pocket costs if participant is randomized to BA) as well as availability for yoga classes or BA sessions. If participants successfully complete the teach-back call, study staff email a link to the baseline assessment via REDCap. Participants deemed eligible based on this baseline assessment are then contacted for enrollment and randomization (see Table 2 for inclusion and exclusion criteria). Participants who do not pass the teach-back are deemed ineligible (see Fig 1).

**Table 2:** Inclusion and exclusion criteria.

*Inclusion*	*Exclusion*
Aged 18 years old or older Experiencing mild to moderate depressive symptoms at study entry (PHQ-9 ≥ 10 and ≤ 20) Able to provide informed consent, are able to read and understand English or Spanish Live in North Carolina, Massachusetts, Minnesota, or Rhode Island Have a healthcare provider whom they could contact if they needed medical care	Have had bone fracture or joint surgery in the past 6 months Are unable to walk Have severe heart failure or lung disease Have had a healthcare provider tell them it is unsafe to exercise Are currently pregnant Are already engaged in study interventions (i.e., yoga practice or psychotherapy more than once in the past 4 weeks or have an intake scheduled for psychotherapy in the next 4 weeks) Have active suicidal thinking (i.e., a positive response to the Columbia Suicide Severity Rating Scale, Screener/Recent–Self-Report screener items 3, 4, 5, or 6), Are currently experiencing possible manic symptoms (Altman Self-Rating Mania Scale score ≥ 6).

Patients are randomized 1:1 using permuted block randomization with blocks of size 2 and 4, stratified by study site. Multiple small block sizes were chosen to reduce the risk of predicting the next group assignment while maintaining overall group balance over the course of the study. A member of the statistical team constructed and uploaded the randomization table to the randomization module in REDCap prior to the start of enrollment. This table assures allocation concealment prior to randomization as members of the research team who perform randomization (by clicking the button “Randomize” in REDCap) do not have access to upcoming group assignments. All study investigators and staff, other than the biostats team, remain masked to allocation sequence throughout the study.

Once study staff reach participants by phone and randomize them, staff inform participants of their randomization condition (YI or BA) and provide an orientation to that intervention. Staff send out intervention-specific materials (i.e., yoga mat and orientation packet for the YI condition, or a journal, pen, and BA orientation packet for the BA condition). The participant then begins the 24-week study. Participants are automatically sent study assessments via REDCap surveys at weeks 3, 6, 9, 12, 18, and 24. In order to minimize missing data, when enrolled participants have not responded for 7 days to automated prompts to complete outcome assessments, study staff who are masked to condition reach out to these participants by phone to encourage them to complete the assessments. Participants have the option to be resent the REDCap link or to complete the surveys over the phone. Participants are compensated up to $170 for completing all study assessments. They are not compensated for intervention participation.

### Interventions

#### Behavioral activation

The 8-session Brief Behavioral Activation Treatment for Depression manual is based on the manual by Lejuez and colleagues [16]. Revisions have been made for length, tele-delivery, the inclusion of bipolar depression in this study, focus on depressed mood rather than major depressive disorder, additional consideration of cultural sensitivity, additional instructions regarding detailed problem-solving of between session activities, inclusion of pleasant activity scheduling that may not be explicitly value connected, and better clarity of procedures for therapists with no background in behavior therapy. These revisions were led by the two BA experts on the team and were informed by several contemporary BA manuals [66–69]. This intervention aims to connect patients with positive reinforcement by facilitating engagement in values-consistent activities that are identified by the individual as being enjoyable and/or important to them. This brief BA treatment involves daily monitoring, values identification, graded activity selection, activity planning, and detailed review of how attempts at activation go in the patient’s life.

All sessions are provided in an individual format via HIPAA-compliant telehealth platforms. BA therapists are mental health clinicians (i.e., LMHCs, MSWs, PhDs) who work in outpatient practices and received expert-led training on the study BA manual. Initial training of each therapist consisted of 7–8 hours of manual review, didactic training, role playing, and conceptualization of example cases.

Given the real-world nature of this trial, unless a person is determined to be uninsured or underinsured, the BA sessions are billed to participants’ insurance. If a participant is uninsured or meets the study definition of underinsured, the study pays for the sessions. Participants are provided with BA worksheets to facilitate their completion of assignments (i.e., daily monitoring, activity planning). All sessions are audio or video-recorded, and participants are always reminded at the beginning of each session that sessions are recorded. As discussed initially during the consent process, at the end of the 8-session treatment (which must be completed within a 12-week period), participants are encouraged to continue to practice BA skills on their own rather than seek additional therapy. However, if there is a clinical need for ongoing therapy during the follow-up phase, including a substantive safety concern, continued therapy with the study therapist is allowed as consistent with real-world clinical practice. Any utilization of healthcare (including therapy during the follow-up period) is tracked to explore its potential impact on study outcomes.

#### Yoga intervention

The 12-session YI is adapted from a yoga instructor manual created by co-Principal Investigator Dr. Uebelacker and colleagues and successfully used in other trials of yoga for depression [51]. All yoga teachers will have completed a Yoga-Alliance approved training program prior to being hired to work on the study. They additionally receive specific training on study procedures and yoga manual. Participants do not pay for yoga classes as there is no pragmatic mechanism to bill classes in the real-world (e.g., bill insurance). All 1-hour yoga classes are provided via a HIPAA-compliant online platform. Synchronous telehealth classes are designed to be physically accessible and gentle for all, especially those with minimal or no prior yoga experience. Participants are asked to have a chair available during the yoga sessions that they may stay seated in or use as a prop (i.e., for balance in standing poses), or choose not to use at all. The yoga teachers offer modifications to postures as needed to meet participants’ individual physical abilities. Thus, classes are intended to be low impact and thus accessible to all regardless of yoga experience or body type. Class structure includes a greeting, brief centering body and breath awareness, warm-ups, energetic and rhythmic practices (e.g., half sun salutations), a series of postures including standing postures, a balance pose, and a twist, and then a final meditation and discussion of personal practice (i.e., practice outside of class); teachers encourage participants to practice yoga between classes. Throughout the class, teachers consistently focus on breath-based movement and mindful attention to the present moment. As with the BA intervention, all yoga sessions are audio or video recorded. To promote additional yoga practice, we provide participants with links to suitable online videos as well as information on how to select other online or community yoga classes.

### Treatment integrity

Treatment fidelity is ensured via standardized and manualized treatment and study procedures, ongoing supervision (i.e., study therapists attend 2 hours of supervision every month and yoga teachers attend 1 hour-long supervision meeting every month) and ongoing therapist/yoga instructor adherence ratings. Fidelity raters rate a random 10% of all recorded sessions to ensure study procedure adherence. Raters use a structured checklist to document fidelity to the respective manuals.

### Measures

**Fig 2:**
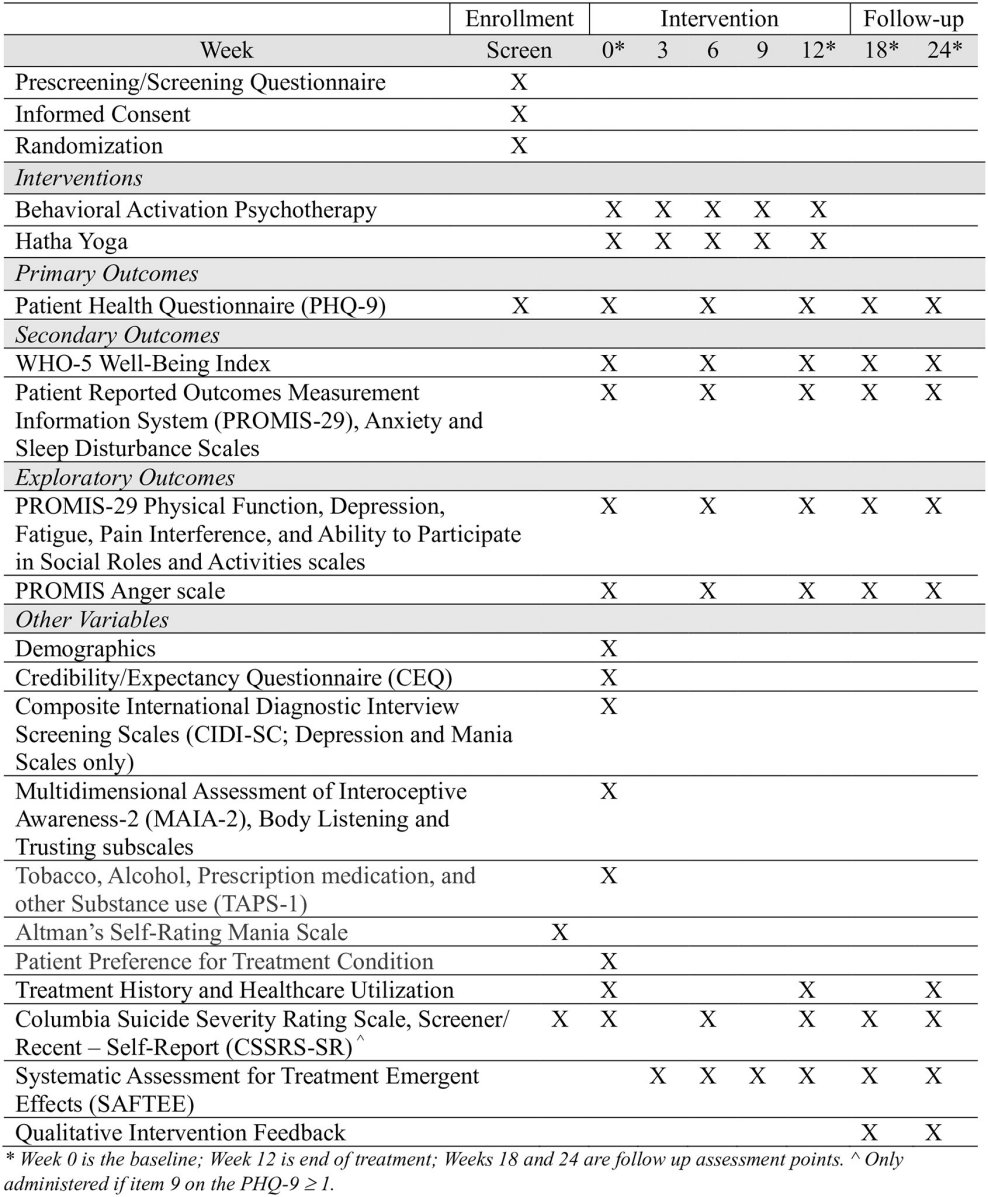
Schedule of enrollment, interventions, and assessments.

Unless otherwise noted, participants complete all measures via self-report in REDCap. (See Fig 2).

#### Primary outcome measure

The primary outcome, depression symptom severity, is assessed using the Patient Health Questionnaire-9 (PHQ-9), which is a 9-item self-report questionnaire [52, 70]. A PHQ-9 score ≥10 has a sensitivity of 88% and a specificity of 88% for major depression. PHQ-9 scores range from 0–27; scores of 5, 10, 15, and 20 represented mild, moderate, moderately severe, and severe depression, respectively [52].

#### Secondary outcome measures

Well-being is assessed using The World Health Organization-5 Well-Being Index (WHO-5) [71, 72], a 5-item self-report questionnaire with positively worded statements related to positive mood, vitality, and general interest over the prior two weeks. The WHO-5 is reliable, has shown strong construct validity as a measure of general well-being, and is sensitive to change [71, 72]. Anxiety and sleep disturbance are assessed using the PROMIS-29 [73], which has seven health domains (physical function, fatigue, pain interference, depression, anxiety, ability to participate in social roles and activities, sleep disturbance). The PROMIS-29 has demonstrated good psychometric properties [73].

#### Exploratory outcome measures

Physical function, fatigue, pain interference, depression, and ability to participate in social roles and activities PROMIS-29 scales are exploratory outcomes. Other exploratory outcomes include substance use, irritability, and treatment utilization. The Tobacco, Alcohol, Prescription medication, and illicit Substance use screening Tool Adapted (TAPS-1) [74] is a 4-item screener adapted from the National Institute on Drug Use and used to measure frequency of tobacco, alcohol, prescription medication, and illicit substances (e.g., cocaine, marijuana, methamphetamine) use. The PROMIS-Anger is a 5-item self-report scale used to measure the exploratory outcome of irritability [75]. A final exploratory outcome will be the number of days of psychiatric inpatient and day hospital treatment in the past 12 weeks, assessed via a self-report questionnaire. These data will be used to examine differences in service usage between the two comparator groups.

#### Safety assessments

The Columbia Suicide Severity Rating Scale, Screener/ Recent–Self-Report (CSSRS-SR) is used to assess suicide-related thoughts and behaviors [76]. The Systematic Assessment for Treatment Emergent Effects (SAFTEE) [77] is widely used in both clinical and research contexts to assess new adverse events over the course of treatment. We will use a modified, self-report version (tailored for the study interventions) that we have successfully used to track adverse events in previous studies [78, 79].

#### Heterogeneity of treatment effect (HTE) variables

HTE variables are used in statistical analyses to understand whether certain subgroup of individuals experience increased benefit from one intervention vs. another. As such, these assessments are administered at baseline only. Participants’ preference for the two treatments, yoga or psychotherapy, is assessed using a single item: “If you had a choice, which program would you prefer to be in?”. Responses range from 1 to 5, where lower scores indicate a stronger preference for BA psychotherapy, and higher scores indicate a stronger preference for yoga [80–82]. The Credibility Expectancy Questionnaire (CEQ) [83] is used to measure treatment expectancy and rationale credibility. It has demonstrated high internal consistency and good test–retest reliability [83]. Finally, the Multidimensional Assessment of Interoceptive Awareness-2 (MAIA-2) [84] assesses the degree to which one experiences one’s body as safe and trustworthy as this may impact one’s comfort and ability to engage in a movement program, such as a YI. The MAIA-2 has demonstrated good psychometric properties [84]. We will use the Body Listening subscale (3 items) and the Trusting subscale (3 items) based on previous research [85–87] and to minimize participant burden. We will also examine the impact of depression severity using the PHQ-9.

#### Sample characteristics

A DSM-5 diagnosis of major depressive disorder or bipolar disorder over the past year is measured by participant responses to the self-report mania/hypomania and major depression sections of the Composite International Diagnostic Interview Screening Scales (CIDI-SC) [74]. The Altman Self-rating Mania Scale (ASRM) [53] is used to assess current manic symptoms. Participants are also asked at baseline to self-report on demographic variables.

#### Treatment utilization

We assess medications prescribed at weeks 0 and 24. Yoga class attendance (including any non-study classes) and amount of personal yoga practice outside of class is assessed by the Yoga Practice Questionnaire [70]. Staff also take attendance at study yoga classes; number of BA sessions completed is tracked by therapist report. For those in the BA condition, the extent to which participants practice the skills they learn in BA outside of therapy sessions is measured by the BA homework record [88], which is completed by the BA therapists and assesses how many activities are scheduled and the extent to which the participant completed each activity.

#### Qualitative intervention feedback

Participants are asked to respond to three open-ended questions about their intervention experience: “How did you use what you learned in the yoga/BA program in your daily life?”, “What got in the way of you using skills from the yoga/BA program in your daily life?”, and “How could we have made this yoga/BA program more helpful to you?” These data will be used inform future trials.

### Statistical analysis

We designed the study to determine the noninferiority of YI versus BA with a margin of 2 points on the PHQ-9 at week 24 (primary outcome). This margin was chosen as its magnitude was below established thresholds of a clinically meaningful difference on the PHQ-9 (which range from 3–5 in the literature) [52]. We arrived at this conclusion based on a few lines of reasoning. One strategy for determining a non-inferiority margin is by calculating it as one half of the mean control effect size from previous trials. A previous study of behavioral activation (BA) vs. cognitive behavioral therapy (CBT) calculated the non-inferiority margin to be 1.9 PHQ-9 points, based on a meta-analysis of previous BA trials that estimated a difference between BA and control groups of 3.8 PHQ-9 points [89]. Another study also choose a margin of 2 based on three pieces of evidence: a) a change of 2 on the PHQ-9 would correspond to a small effect size (assuming a standard deviation of 6.9), which is typically seen to be of limited clinical value; b) 2 points on the PHQ-9 represents an effect size of less than one half the expected difference between treatment and usual care; and c) psychologists using the PHQ-9 in clinical practice did not see a change of less than 2 points on the PHQ-9 to be a change that was clinically important [90]. A margin of 2 points on the PHQ-9 has also been used in prior noninferiority trials of depression treatments [91–96]. We include the non-inferiority margins for secondary outcomes in Table 3.

**Table 3:** Non-inferiority margins and power estimates for primary and secondary outcomes.

Outcome Type	Description	Measure	Non- inferiority Margin [Supporting Citations]	Anticipated Standard Deviation
Primary	Depressive symptoms	PHQ-9^a^	2 points^d^	7 points
Secondary	Well-being	WHO-5^b^	1.5 points [95, 109]	5 points
Secondary	Anxiety	PROMIS-29^c^	3 points [92–94]	10 points
Secondary	Sleep Disturbance	PROMIS-29^c^	3 points [92–94]	10 points

^a^ Range of possible scores = 0–27.

^b^ Range of possible scores = 0–25.

^c^ Converted to t-scores, i.e., with a mean = 50 and SD = 10.

^d^ See text for supporting citations.

Assuming an actual mean difference of zero, a standard deviation of 7 in both arms [97, 98], and a 25% dropout rate by week 2 [33], a sample size of 518 (259 participants per arm) provides 80% power to establish the noninferiority of the YI versus BA based on a one-sided 2.5% significance level t-test against the margin of -2 points.

All statistical reporting will adhere to guidelines in the CONSORT extension for noninferiority trials [99]. The primary outcome is patient-reported depression symptom severity (PHQ-9) at week 24. The difference in the mean scores at week 24 will be estimated using a linear mixed effects model with random participant intercepts and slopes and fixed effects for intervention arm, study site, time in weeks, and intervention-by-time interaction. Natural cubic splines or categorical time regression terms will be used if mean trajectories are nonlinear. The non-inferiority of the YI will be established based on a one-sided 2.5% significance level test of the estimated difference in means against the prespecified margin of 2 points. We will also report corresponding model-based estimates with 95% confidence intervals (CIs).

Secondary outcomes including well-being (WHO-5), anxiety, and sleep (PROMIS-29) will be analyzed similarly using linear mixed models, one-sided noninferiority tests against the prespecified margins, and model-based estimates with CIs. A multiplicity correction will be used to interpret secondary outcome results to maintain an overall family-wise error rate of 5%. Exploratory outcomes will be analyzed using linear mixed models or other appropriate longitudinal regression models (e.g., quasi-poisson generalized estimating equation model for number of days of psychiatric inpatient treatment); exploratory results will emphasize model-based estimates and CIs.

To examine potential heterogeneity of treatment effect (HTE), we will include participant-level baseline variables as moderators in the primary outcome model for PHQ-9 described above. Prespecified moderators include intervention preference, perceived intervention credibility, trust in one’s body, and depression severity. For each potential moderator, the linear mixed effects model for the PHQ-9 described above will be expanded to include an additional fixed effect for each moderator, a three-way moderator-by-intervention-by-time term, and all lower-order two-way interactions. HTE will be formally evaluated using a likelihood ratio test of the three-way interaction term, a term representing the estimated differential intervention effect across levels of the moderator. Model-based estimates, 95% CIs, and p-values will be presented descriptively using tables and figures (e.g., a forest plot). Additionally, we plan to explore the role of demographics, clinical diagnoses, and treatment history as potential moderators. We will follow a similar HTE analytic strategy for these post hoc exploratory moderators and report model-based estimates with 95% CIs.

Our primary linear mixed models incorporate all available longitudinal data from participants including those that drop out of the study prior to week 24. Sensitivity analyses will be conducted by further adjusting regression models (and, thus, model-based estimates and CIs) for any baseline imbalances between intervention groups or any variables known or suspected to be associated with dropout or missingness. We will also provide thorough summaries of reasons for missingness, proportions of missing data, and differences in the characteristics of patients with and without missing data.

### Study oversight

#### Centralized IRB and Data and Safety Monitoring Board (DSMB)

This study utilizes a centralized IRB such that the study sites have ceded review to the Mass General Brigham IRB (the “reviewing IRB”). The DSMB, which meets via teleconference every 6 months, is responsible for ongoing monitoring of the trial with the purpose of ensuring study safety and integrity. The DSMB members, as well as an internal safety monitor with expertise in clinical trials of depression, review any serious adverse events that are possibly, probably, or definitely related to study participation. The DSMB prepares an annual report of its findings regarding safety and quality with any recommendations to improve patient safety, protocol adherence, or data quality. This report is sent to the Mass General Brigham IRB.

#### Study committees

Our study committees are described in Table 4. We highlight here the role of our Advisory Board. Study partners (i.e., members of the Advisory Board) are involved in all aspects of study design, implementation, and dissemination of study findings. The Advisory Board meets at least quarterly, but also more frequently as needed (e.g., monthly prior to launching the study). Our partners hold expertise and experience of relevance to the current proposal, and include individuals with lived experience of depression, caregivers for an individual(s) with lived experience of depression, yoga instructors, mental health providers, payors (e.g., representatives from insurance companies), experts in clinical trials of depression, diversity, inclusion, and equity experts, and representatives from advocacy organizations. The Advisory Board discusses key study decisions and makes recommendations to the Steering Committee, which then provides direction and feedback to the rest of the study committees. For example, some partners are experts in patient experience and retention in clinical research and they can offer specific guidance on improving recruitment strategies. Partners also offer guidance on the format and content of outreach efforts to aid recruitment of individuals across racial/ethnic backgrounds, education, and income levels and to keep participants engaged in completing the study interventions and assessments.

**Table 4:** Description of study committees.

*Committee*	*Description*
Steering Committee	Responsibilities: Oversee all study committees and implementation of study procedures at all sites. Provide input on key scientific issues that arise during study implementation, operation, and data analysis phases. Members: Study PIs, site PIs and co-Is, Safety Officer, site research managers, study partners. Frequency of meetings: Biweekly (every other week).
Study Operations Committee	Responsibilities: Write manual of procedures; support implementation of study operations across sites. Review adverse events, protocol deviations, participant safety, and recruitment and retention. Members: Study PIs, site research staff Frequency of meetings: Weekly.
Communications Committee	Responsibilities: Oversee creation of study recruitment and engagement materials. During study operation, discuss ways to increase recruitment and engagement (i.e., ways to distribute social media advertisements, advertisement language) with Advisory Board, study staff, site PIs, and study PIs. Collaborate with Data Committee and Steering Committee on dissemination of results. Members: Co-Is, communication manager, one staff member from each site, social media consultant, and study partners. Frequency of meetings: Biweekly during study start-up, and then as needed once study is running.
Data Committee	Responsibilities: Oversee database creation and management, ongoing data integrity checks, and data analysis and interpretation. Members: Data managers, statisticians, study PIs, staff from each site who regularly use the REDCap database. Frequency of meetings: Biweekly.
Yoga Intervention Committee	Responsibilities: Oversee training, implementation, and fidelity ratings of yoga intervention. Provide supervision to yoga teachers. Members: Yoga consultants/fidelity raters, a PI, co-I, study partners with expertise in YIs, and study staff who help to host yoga classes. Frequency of meetings: Monthly during study start-up, then bimonthly.
Behavioral Activation Intervention Committee	Responsibilities: Oversee training, implementation, and fidelity ratings of psychotherapy sessions. Host BA therapist supervision meetings. Members: Co-Is and study partners with expertise in BA. Frequency of meetings: Biweekly.
Data Safety and Monitoring Board (DSMB)	Responsibilities: Review adverse events, protocol deviations, data integrity, recruitment and retention, and participant demographics. Members: Four doctoral-level individuals not otherwise involved in this study and independent from PIs and co-Is. Frequency of meetings: Twice per year.
Advisory Board	Responsibilities: Advise Steering Committee on design and implementation of the study and dissemination of study results. Members: Study partners, including people with lived experience, caregivers, yoga instructors, mental health providers, payors, patient advocates, and people with expertise in other relevant areas (e.g., study design, dissemination). Two members of the Steering Committee attend. Meeting frequency: Quarterly or more often as needed.

### Safety procedures

#### Procedures for monitoring adverse events

Adverse events (AEs) as defined by OHRP [100] are assessed in two ways: 1) the participant mentions an experience during an intervention session that the instructor/clinician records as a potential AE; or 2) the participant self-reports a potential AE (e.g., a scheduled surgery, muscle soreness) via a survey question. All potential AEs are reviewed by the research team and coded for severity, relatedness, and expectedness. AEs are discussed on the weekly Internal Study Operations and Safety committee meeting with staff from all study sites. Serious adverse events (SAEs) are reviewed immediately (within 24 hours) by the study PIs and/or the internal safety monitor and reported promptly to the IRB, DSMB, and PCORI (if required). All SAEs and related AEs are shared with the IRB, DSMB, and PCORI in annual progress reports.

#### Procedures for monitoring suicidal ideation

Suicidal ideation and behavior are assessed every 6 weeks via the CSSRS Screen Version–Recent SR in REDCap. If a participant’s scores indicate suicidal ideation or suicidal behavior in the past 3 months (i.e., positive response to items 3, 4, 5, or 6), the participant receives an automated REDCap message with a) information about the National Suicide Prevention Lifeline (988) and associated website (the Lifeline is a 24/7 hotline that provides help for those at risk for self-harm), b) encouragement to reach out to a local healthcare provider, and c) a telephone number for study staff who can connect them to a study clinician. If a participant specifically endorses thinking about a method for suicide, reports an intention to act, reports preparatory behaviors, or reports an attempt in the past 3 months, REDCap provides an additional message to the participant informing them that a study clinician will outreach by telephone within the next 24 business hours. Concurrently, an automated message is sent to the Principal Investigators and study staff informing them to reach out to the study participant. A study clinician then initiates the outreach phone call, conducts a suicide risk assessment, and offers support/resources as needed. We use similar procedures if a person verbally reports suicide ideation to study staff outside the context of an assessment. That is, study staff will remind the participant about 988 as well as contact the covering clinician who will assist with an appropriate follow-up plan which may include a risk assessment with the participant. If the participant is at immediate risk, study staff will call 911 to send help to the participant’s address prior to calling the covering clinician. If a randomized participant requires a higher level of care (e.g., additional outpatient services, inpatient or partial hospitalization), they may remain in the study if they wish and may or may not continue with the study interventions depending on what is determined to be clinically appropriate by the participant and their providers. All additional services will be tracked as part of study assessments.

## Design considerations for a pragmatic effectiveness trial

Given existing research supporting the efficacy of yoga interventions and BA for depression, we determined that a pragmatic, effectiveness clinical trial was the next step in understanding how yoga interventions might have an impact on depression in the community. Therefore, we made several study design decisions to reflect a pragmatic, effectiveness trial [101]. For example, we made eligibility criteria as broad as possible, including people who may have bipolar disorder or who are taking medications for depression, and we required that participants have elevated depression symptoms but not a diagnosis of major depression for participation. The large sample size should increase the chances that study groups are ultimately balanced on these key variables.

Second, we selected BA therapists who are engaged in clinical practice (i.e., not research therapists) and have a wide range and amount of prior experience and training in CBT or BA approaches. Moreover, the BA therapists bill study participants’ insurance for participants who have adequate insurance to mirror real-world activity. The yoga intervention is also delivered as it might be in the real world, via a telehealth synchronous class with trained yoga instructors. Also, in line with a pragmatic trial, we provide interventions as they are typically delivered in the community: individually for BA and in a group format for yoga (i.e., typically 5–10 people per class). Finally, we selected a primary outcome, the PHQ-9, that is commonly used in clinical practice to assess improvement in depression symptoms.

As a result of this pragmatic study design, there are some key differences in the delivery of the two study interventions that may ultimately have an impact on overall participant adherence or satisfaction. First, the length of time to intervention start may differ. Because this is a study, we have made significant efforts to ensure that participants can start BA within 1–2 weeks of randomization; however, for scheduling and other reasons, this timing may not always occur. A participant can always start a yoga class in their first week after randomization. Second, another key difference in the interventions is that yoga classes are offered at 4 set times each week in a group format, whereas the BA sessions are determined based on the participant and therapist schedules, which may or may not allow for flexibility in scheduling. A third difference is that the BA participants often incur a cost per session given that most insurers require a co-pay for each BA session whereas the YI is delivered with no costs to participants. To increase accessibility of the research, we capped the out-of-pocket cost for the BA sessions at $25 out-of-pocket per session but nonetheless, the cost may be a burden to some participants. In contrast, yoga is provided at no cost to participants given that it is often not currently covered or reimbursed by insurers and to reflect that there are opportunities for free synchronous and asynchronous online classes in the community. For example, one insurer, Blue Cross Blue Shield (BCBS) of RI, offers free classes to its members at a BCBS store. Other community organizations, such as the YMCA, offer low-cost memberships which include yoga classes. A final key difference between the yoga and the BA interventions is documentation in the medical record–yoga classes are not documented in any medical record, whereas psychotherapy, or BA, must be documented to be reimbursed. As a result of these differences, study results will reflect potential outcome differences not only based on the “pure” intervention, but the interventions as they are delivered in the real-world, including several barriers to mental health treatment (i.e., access to BA) and access to potentially helpful treatments such as yoga.

Consistent with PCORI’s goal for pragmatic clinical trials to maximize reach [50], we employ specific strategies to ensure that our procedures are accessible to a wide range of patients. For example, we tailor recruitment materials and interventions for Spanish-speaking individuals. Participants have the option to engage in BA in Spanish with a Spanish-speaking therapist or join yoga classes with a Spanish-speaking instructor. This tailoring may help overcome language barriers that can impact enrollment of Spanish-speaking participants in clinical trials [102]. Our decision to offer the study in an entirely virtual format was chosen to further maximize accessibility and to limit barriers to study participation (i.e., cost and time associated with travel) [103]. However, we are aware that some participants may not own smartphones or computers, and so we provide tablets to participants as needed to ensure that lack of electronic device ownership is not a barrier to study entry.

This pragmatic effectiveness trial is not an implementation trial [104]. Therefore, some aspects of this study are less pragmatic and more consistent with a research study opposed to full real-world implementation. For example, we use interventionist manuals for both yoga teachers and BA therapists and have procedures to ensure treatment integrity (e.g., we provide therapist/teacher manual training, require ongoing supervision meetings, and conduct assessments of interventions delivered). We created manuals that focus on core intervention elements, but also allow some flexibility in how the interventions are delivered. Such flexibility allows interventionists to tailor the intervention to participant(s) which is more consistent with real-world practice.

## Lessons learned

We launched study enrollment on February 3, 2022; as of September 1, 2024, we have screened 1558 participants and randomized 320 participants across four sites. To date, we have not made any substantive changes to the protocol; however, in conducting a pragmatic study comparing virtual yoga to BA, we have learned some important lessons. First, we have learned that there are challenges with requiring out-of-pocket payments (i.e., to cover BA sessions) from participants, even though this is consistent with real-world practice. As such, the study will cover the costs of BA for individuals who do not have insurance or who are underinsured (defined as having an out-of-pocket cost for BA sessions > $25/session). We also learned the importance of being transparent with the potential insurance costs, such that participants knew exactly what their potential payments for BA would be ***prior to*** being randomized. Providing this information requires additional effort for study or clinic staff to ascertain insurance benefits for *all* eligible participants (opposed to those just randomized to BA) but seemed necessary given participant questions and concerns about cost. Despite this transparency and covering costs for those who are under insured, we had 27 participants opt not to enroll in the study due to concerns for covering their insurance co-pays if randomized to BA.

Second, we have learned the importance of investing in social media to assist study recruitment efforts as we have found that most participants screened thus far (i.e., 764 of 1558) and ultimately, eligible for the study (i.e., 320) were identified through social media. As such, social media has greatly helped us to consistently meet our monthly recruitment goals (i.e., randomize 13 participants/month across the 4 sites). Social media outreach has also helped us towards meeting our goals for representation from racial, gender, and ethnic groups in the study sample. as we have been tailoring social media posts for specific groups underrepresented in clinical trials for yoga. We have spent approximately $2300/month on average across the four sites to support outreach as well as engaged a social media consultant to help us establish our social media campaign. This consultant, along with experts on our team, used several strategies to optimize the campaign, such as using more images than words and imprinting the words on the image, tailoring social media posts to specific zip codes, segmenting the campaigns into three age groups (18–35, 36–50, and 51+), and employing diverse language and images relevant to different groups. We also developed culturally tailored advertising materials in Spanish to help recruit Spanish-speaking populations. We learned it is important to not completely stop and then re-start a social media campaign as a social media outlet (e.g., Facebook and Instagram) has algorithms that will help to refine and optimize the campaign, but this customization is lost if you stop a campaign. Therefore, it is better to spend a minimal amount each month to keep it running than stopping it completely.

In conducting this large, pragmatic, online study, we have observed that there are individuals who do not have an intent to join the study, but still begin the screening and/or consent process for the study, potentially in the hope of earning money for completing assessments. We have labeled these participants as “potentially fraudulent.” These individuals are discovered and labeled as such often due to a constellation of factors, such as: a) they provide fake addresses or out-of-service phone numbers; b) they refuse to talk to us by phone; c) they enter odd names (Nike, Wine); or d) or respond to the screening questionnaire with “straight line” assessments (i.e., endorse one consistent number or response across all assessments or have no variation in their responses). In conducting the study, we have observed particular months that have had spikes in potentially fraudulent participants and suspect that this may be due to certain websites or blogs sharing the study as an opportunity to make money.

Minimizing potentially fraudulent participants is important for study integrity. As such, there are several strategies to prevent these participants from being enrolled and randomized. First, we have found that it is very important that study staff speak with each potential participant prior to enrollment as well as perform a “teach-back” (structured questions about the study procedures to ensure that the participant understands). Unfortunately, using a captcha (e.g., a program intended to distinguish human from machine input) will not dissuade these participants because they are human; it will only prevent bots from entering data. There are different perspectives on paying study participants for completing study assessments and advertising such payments, but most studies do offer some monetary compensation [105–107]. We offer compensation for assessments and although not advertised through our social media campaign, it is noted on our study website and therefore is publicly available information. We believe that this information about the opportunity to be paid for completing online assessments did contribute to our study being accessed by potentially fraudulent participants. Thus, there may be a tension between sharing necessary study information and individuals using this information in unintended ways.

Other lessons learned in conducting this study include the importance of offering evening yoga classes and immediate scheduling of the BA sessions. We observed more interest in our evening yoga classes likely given participants’ other commitments during the day and thus, we offered two evening classes. When the study first started, in the BA group, we also observed some long delays (e.g., a month) before the first therapy session or attrition with participants not attending any sessions at all. At that time, we were relying on the BA clinicians to follow up with participants to set-up the first session post-randomization. We changed procedures so that study staff could schedule an initial BA session while on the phone with a participant immediately after randomization. This shift led to reduced attrition as well as a shorter time to first session after randomization.

## Conclusion

There are many strengths to this study. All interventions in this study are manualized [16, 51], and clinicians/instructors are provided with formal training prior to commencing the intervention. Regular assessments of treatment fidelity (i.e., review of recordings) ensures that the interventions continue to be administered consistent with these manuals. Similarly, study staff across all sites meet weekly and share a detailed Manual of Procedures to ensure that procedures are similar across sites and over time. We offer the study in both English and Spanish, and we make efforts to reduce economic barriers to study participation and maximize reach. Lastly, as described previously, our study’s rigor is enhanced by incorporation of perspectives from our community partners and other committee members who provide essential insight on study recruitment materials, study safety concerns, study interventions, study operations, and study data, among other key topic areas.

Our clinical trial also has limitations. The centralized IRB process, though a beneficial inclusion, did have an impact on the timeliness of study start-up. In addition, the entirely virtual format of the study may be challenging for specific populations (e.g., older adults who may experience difficulties with using technology or hearing audio, though across studies older adults do report treatment satisfaction with telehealth comparable to in-person care) [108]. The multi-step nature of the study screening process may also be a deterrent to participation for some individuals; this process was designed to provide participants with as much advance information to inform their participation and maximize efficiency (i.e., by incorporating online pre-screen questionnaires).

Despite these limitations, our trial’s many strengths make it well-equipped to evaluate the real-world comparative effectiveness of two efficacious treatments for depression. Our trial will help us answer whether the YI is non-inferior to BA, an established treatment for depression, as well as identify individual characteristics that predict a better response to one treatment or the other. Depending on the results, it is our hope that we can have a direct impact on clinical practice guidelines inclusion of YIs as an intervention for depression.

## Supporting information

S1 File Institutional review board intervention/interaction detailed protocol. (DOCX)

S1 Checklist SPIRIT 2013 Checklist: Recommended items to address in a clinical trial protocol and related documents*. (DOCX)
