# Supplementary material for: Expanding first-line options for depression: Protocol of a pragmatic comparative effectiveness trial of yoga vs. behavioral activation (the COMPARE study)
Source: PLoS One. 2025 Jan 6;20(1):e0315506. doi: 10.1371/journal.pone.0315506 (PMC11703115; doi:10.1371/journal.pone.0315506)
Supplement: S1 File — (DOCX) [file pone.0315506.s001.docx]

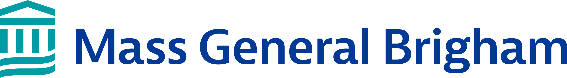


# Institutional Review Board Intervention/Interaction Detailed Protocol

Principal Investigator: Louisa G. Sylvia, PhD / Lisa Uebelacker, PhD

Project Title: COMPARE: Expanding first-line options for depression and matching treatments to patients: hatha yoga vs. behavioral therapy

Version Date: 2/12/ 2023

Version Name/Number: 6

*For Intervention/Interaction studies, submit a Detailed Protocol that includes the following sections. If information in a particular section is not applicable, omit and include the other relevant information.*

# Background and Significance

Major Depressive Disorder is a highly prevalent mental health problem that results in substantial suffering, impairment, and financial burden. Further, clinically significant depression symptoms that may not meet full criteria for major depression are prevalent and are associated with decreased quality of life[^1^](#_bookmark0)[^,2^,](#_bookmark1) increased risk for major depression[^3^,](#_bookmark2) increased economic costs[^4^](#_bookmark3), and even increased mortality[^5^.](#_bookmark4)

American Psychological Association (APA) guidelines recommend either an evidence-based psychotherapy (e.g., behavioral or cognitive-behavioral therapy) or pharmacotherapy as a first-line treatments for adults with depression[^6^.](#_bookmark5) However, because these first-line treatments are often not ideal for reasons described next, there is a need for alternative treatments for depression. First, people with depression are increasingly interested in physical activity[^7^](#_bookmark6) and complementary and integrative health interventions including mind-body approaches for treating depression[^8^.](#_bookmark7) In particular, approaches such as **yoga** are recommended for depressed adults for whom first-line treatments are either ineffective or unacceptable[^6^.](#_bookmark5) Second, many patients experience barriers to first-line treatments including cost, challenges with access, reluctance to disclose personal information, concerns about stigma and privacy, or medication side effects[^9^.](#_bookmark8) Third, first-line treatments do not work for everyone, even when delivered by expert clinicians[^10^.](#_bookmark9) In sum, whereas current first-line treatments for depression are acceptable and helpful for many adults with depression, there is increasing interest in evidence-based integrative health interventions, such as yoga, for treating depression and improving overall well-being.

Therefore, we seek to answer the following crucial question (or health decision): Could yoga be considered a first-line treatment for depression? We plan to compare real-time, online yoga classes to a first-line treatment for depression, namely, behavioral therapy (also provided remotely via video- or telehealth), in a non-inferiority trial. The results of this study will inform providers, patients, payors, and advocates as to whether yoga is as effective as another first-line treatment for depression, therefore building the case that yoga should be supported financially by health insurance. We also seek to answer a second question: Who is more likely to benefit from yoga and who is more likely to benefit from behavioral therapy? We will answer this question using heterogeneity of treatment effect (HTE) analyses

that can determine if certain subgroups of depressed patients improve more with one treatment or the other.

Yoga is an effective depression treatment and is widely available and commonly used. In the United States (US), most yoga that is practiced is *hatha* yoga, a form of yoga in which physical postures are used in addition to meditative practices. Hatha yoga has been called “meditative movement.” Many yoga classes (including the Kripalu style of hatha yoga that we will use in this study) include mild- intensity physical activity[^11^](#_bookmark10) as well as instruction on non-judgmental attention to present moment experiences (i.e., mindfulness practices). One important mechanism by which yoga may have an impact on depression is cultivation of mindfulness skills for use in daily life. There are many reasons to study the non-inferiority of yoga compared to an evidence-based psychotherapy for depression.

First, recent meta-analyses have documented yoga as an efficacious and well-tolerated treatment for depression[^12^](#_bookmark11)[^,13^,](#_bookmark12) although they also cite the need for more high-quality research. Second, yoga classes focus on overall well-being as opposed to symptoms and thus, could be more appealing to people concerned with the stigma of conventional mental health treatment[^14^.](#_bookmark13) Indeed, the popularity of yoga has increased dramatically in the U.S.: 14.3% of the population reporting engaging in yoga in 2017 compared to 9.5% in 2012[^15^.](#_bookmark14) In fact, a 2020 Pew research poll found that 8% of U.S. adults engaged in yoga at least weekly and 12% at least monthly to cope with stress from the COVID pandemic[^16^.](#_bookmark15) Third, yoga may alleviate somatic problems that are often comorbid with mood disorders (e.g., back pain[^17-19^,](#_bookmark16) osteoarthritis[^20^](#_bookmark17)). Fourth, online, synchronous (live-streaming) and asynchronous (pre-recorded) yoga is widely available, with numerous free asynchronous classes and opportunities for low-cost synchronous classes[^21^](#_bookmark18)[^,22^.](#_bookmark19) Moreover, during the COVID pandemic, with many gyms and yoga studios closed, yoga teachers vastly expanded their online offerings[^23^](#_bookmark20)[^,24^,](#_bookmark21) and there was a large increase in the number of students who participated in online classes[^23^](#_bookmark20)[^,25-27^,](#_bookmark22) with forecasts that this increased online presence would continue after the COVID crisis subsided[^26^.](#_bookmark23) Fifth, recent data highlight the efficacy and acceptability of online yoga programs. For example, Huberty et al. found that a moderate amount of online yoga was superior to a stretch-and-tone intervention in decreasing depression symptoms for women with perinatal loss[^28^.](#_bookmark24) Data from the VA found that yoga delivered via either telehealth or in person was acceptable and associated with improvements in health[^29^.](#_bookmark25)

A final important reason to examine yoga as a treatment for depression is that yoga is not currently considered a first-line treatment option for depression and is not typically paid for by insurance[^30^.](#_bookmark26) Per our discussion with insurance stakeholder Ms. Marootian, Director Strategy and Business Development, Neighborhood Health Plan (see Letters of Support), treatment guidelines from national organizations (e.g., American Psychological Association; US Preventative Services Taskforce) provide an important source of information for insurance companies to inform which interventions should be covered. A large-scale trial that supports the effectiveness of yoga for depression, combined with other, smaller, recent studies, could lead to the next update to treatment guidelines recommending yoga as a first-line treatment for mild-moderate depression. There are two lines of argument to support this. First, treatment guidelines for depression already discuss physical activity and yoga as treatment options. The National Institute of Clinical Excellence guidelines in the UK suggest “low intensity psychosocial interventions,” including a “structured group physical activity programme” as first-line treatment for people with mild-moderate depression[^31^.](#_bookmark27) The APA guidelines for treating

depression discuss use of physical activity as well as yoga: “For adults with depression for whom psychotherapy or pharmacotherapy is either ineffective or unacceptable, the panel suggests exercise monotherapy [i.e., exercise alone] or St. John’s Wort monotherapy. If neither is acceptable or available, the panel suggests consideration of… yoga”[^6^.](#_bookmark5) Second, the importance of yoga as a currently available healthcare intervention is notable in the Veteran Administration (VA) Medical Centers. Since mandated in 2016, the VA has expanded complementary and integrative medicine offerings across the country,

focusing on evidence-based interventions to improve pain, mental health, and well-being. A survey found that yoga, along with meditation and relaxation interventions, is one of the top five most frequently offered approaches in the VA system[^32^.](#_bookmark28) Thus, this study could have direct and immediate public health significance.

**Behavioral Activation (BA) is effective for treating depression and is widely used in community settings.** We selected BA as a comparator for the following reasons. First, it is a specific type of behavioral therapy that is a first-line treatment for depression per APA treatment guidelines for depression[^33^.](#_bookmark29) Second, large-scale randomized controlled trials of BA have demonstrated its efficacy and cost-effectiveness for depression[^34-36^.](#_bookmark30) Third, BA strategies are commonly used by community therapists[^37^,](#_bookmark31) and BA strategies are considered to be easier to teach to therapists than more complex full suite of CBT interventions[^38-40^.](#_bookmark32) Fourth, extensive evidence indicates that synchronous video and telephone-based therapy is effective,[^41-44^](#_bookmark34) including a recent meta-analysis of telephone-administered psychotherapy for depression[^45^.](#_bookmark35) There are data from RCTs specifically documenting the effectiveness of remotely delivered BA in various populations as well[^46^](#_bookmark36)[^,47^.](#_bookmark37) Finally, insurers are covering telehealth for psychotherapy, due in part to the COVID pandemic.[^48^](#_bookmark38)[^,49^](#_bookmark39)

In sum, we need better treatment for depression and it is unclear which treatments are best for whom*.* Depression is a heterogeneous entity, and choice of treatments frequently occurs based on clinician or patient preference and using trial-and-error[^50^.](#_bookmark40) Even with efficacious treatments, many individuals do not respond, or have only a partial response, to an initial treatment for depression. Thus, there is need to develop a science of personalized medicine, in which characteristics of an individual can help to predict which treatment will be the most efficacious for that individual.

# Specific Aims and Objectives

The first goal of the proposed research is to determine whether a hatha yoga program for depression is non-inferior to BA in reducing depressive symptoms over a 6-month period. Our second goal is to identify individual-level characteristics that predict heterogeneity of treatment effect (HTE) to develop guidance for matching patients to the optimal depression treatment.

## Specific Aim #1: To compare the effectiveness of synchronous virtual yoga and telehealth BA in treating depression over 6 months.

**Hypothesis 1a**: Yoga will be non-inferior to BA on the primary outcome of depression symptoms at 6 months.

**Hypothesis 1b:** Yoga will be non-inferior to BA on the secondary outcomes of overall well-being, anxiety, and sleep at 6 months.

## Specific Aim #2: To determine participant-level characteristics that predict heterogeneity of treatment effect (HTE) over the 6-month study period to better match participants to treatments.

**Hypothesis 2:** There will be greater effectiveness of yoga compared to BA among participants with a stronger preference for yoga, whereas there will be greater effectiveness of BA compared to yoga among participants with a stronger preference for BA.

**Hypothesis 3:** There will be greater effectiveness of yoga compared to BA among participants with higher perceived credibility of yoga relative to BA, whereas there will be greater effectiveness of BA compared to yoga among participants with higher perceived credibility of BA relative to yoga.

**Hypothesis 4:** There will be greater effectiveness of BA compared to yoga among participants with more severe depression at baseline.

**Hypothesis 5:** There will be greater effectiveness of yoga compared to BA among participants with a higher degree of trust in their bodies.

# General Description of Study Design

This is a parallel group study design. We propose to compare an online, synchronous group- based hatha yoga intervention developed for individuals with depression70 to an online, synchronous individualized, evidence-based behavioral therapy for depression, or Brief Behavioral Activation Treatment for Depression (BA)[^51^.](#_bookmark41) This study is a multi-site randomized trial of adults with clinically significant depressive symptoms. Participants (N=518) will be randomized in an equal allocation ratio (i.e., 1:1) across two intervention groups: yoga and BA. Interventions will be provided over a 12-week period and assessments will occur at baseline (week 0), week 6, week 12, week 18, and week 24.

# Subject Selection

## Inclusion criteria:

- 1. Aged 18 years old or older
  2. Depressive symptoms at study entry (PHQ-9≥10)
  3. Provides informed consent
  4. Able to read and understand English or Spanish.
  5. Live in NC, MA, MN, or RI
  6. Has a healthcare provider (a primary care provider, clinic, or mental health care provider) whom the participant could contact if medical care were needed

## Exclusion criteria:

1. PHQ-9 > 20
2. A bone fracture or joint surgery in the past 6 months
3. Unable to walk
4. Severe heart failure or lung disease
5. Had a healthcare provider tell them it is unsafe to exercise
6. Currently pregnant
7. Are already engaged in study interventions (i.e., engaged in yoga practice or psychotherapy more than once in the past 4 weeks or has an intake scheduled for psychotherapy in the next 4 weeks).
8. Have active suicidal thinking (i.e., PHQ-9 item 9 ≥1 and a positive response to CSSR-S screener items 3, 4, 5, or 6 [Past 3 months])
9. Are currently experiencing manic symptoms (Altman Self-Rating Mania Scale score ≥ 6).
10. Unable to complete study procedures (i.e., attend yoga classes, BA sessions)

**NOTE REGARDING SPANISH-SPEAKING PARTCIPANTS:** All study materials (e.g., advertisements, description of the study, consent forms, assessments, intervention materials) will be available in English and Spanish. Each site recruiting Spanish-speaking patients will have Spanish-speaking staff and interventionists to work with those patients.

## Recruitment

**How individuals are identified for recruitment.** Participants will be recruited from diverse sources with extensive reach, including

- - Affiliated partnering organizations (e.g., Depression and Bipolar Support Alliance; National Alliance, National Alliance on Mental Illness, Anxiety and Depression Association of America)
  - Social media sites and online publications
  - Primary care and psychiatry clinics, and other community-based clinic settings
  - Direct referrals from community providers who will speak with the potential participant about the study prior to referring them to study staff
  - Via electronic health records (EHR), using methods approved by local sites. Study staff at each site will run reports to determine who is eligible in their EHR based on the primary enrollment criteria (e.g., age>18 yo, speak English or Spanish). Study staff (e.g., most often clinical research coordinators) will then contact them (e.g., via email, text, phone, mail) with IRB-approved study recruitment materials (e.g., flyers, emails about the study, prepared scripts describing the study) to determine whether they are interested in this study.
  - Contact participants in the MoodNetwork Data Repository (IRB Protocol #2014P001346) #2014P001346) who have previously consented to receive new study information.
  - Via Research Match ads (Vanderbilt recruitment tool)

All advertisements will be IRB-approved.

Participants who are interested in the study and identified by any of the recruitment means described above will be emailed a link to the REDCap database, including the consent form, which will provide information about the study as well as a way to contact study coordinators by phone with questions.

Participants will be recruited on a rolling basis.

## Sample

Our sample will be representative of the target population: individuals with depression. There are several aspects of the current study design that we believe will enhance recruitment in diverse patient populations.

- - Both study interventions will be offered via telehealth to reduce barriers to access (e.g., childcare, time, parking/transportation costs, less time out of work).
  - We have study funds to cover the costs of the technology for people who do not have a device or home-based internet access. Further, to decrease barriers related to digital literacy, study staff will be proactive about assisting participants with setting up technology, using technology and troubleshooting if needed.
  - We have study funds to pay for the BA therapists for participants who are uninsured or underinsured.
  - We have study funds to offer the yoga intervention free of charge to participants.
  - We will offer study participation to Spanish-speakers.
  - We have an Advisory Board of stakeholders with diverse backgrounds (i.e., racially and ethnically diverse as well as diversity in socioeconomic status, educational background, and clinical features) to assist with the implementation and oversight of this study. We will recruit participants through the EHR to expand the potential reach of study engagement materials, specifically to under-represented minorities in clinical research.

# Subject Enrollment

Interested participants will complete a pre-screening questionnaire which asks about the following eligibility criteria: state of residence, age, medical conditions, ability to walk, pregnancy, and engagement in psychotherapy. There is no consent for the pre-screening questionnaire. If it seems that participants are potentially eligible, they will be given the contact information of study staff to discuss the study further. If they are still interested in the study after the pre-screening questionnaire as well as after speaking to study staff (if they choose to do this), we will send them a link to REDCap to complete an information sheet (i.e., informed consent form with a checkbox agreeing to participation).

Once they consent, participants will complete a screening questionnaire in REDCap to further determine eligibility. This questionnaire assesses all remaining inclusion criteria: current engagement in psychotherapy or yoga classes; depression symptoms (via PHQ-9), suicidality (via CSSRS), and mania (via Altman Mania Scale). If the participant appears eligible, they will be asked to have a telephone conversation with study staff to review the study. Study staff will document that participants’ questions about the study were answered and that participants remained interested in the study.

If fraud is detected within the surveys, the Principal Investigators may decide to withdraw a participant from the study. Fraud may include 1) a research assessment being completed in an unusually short period of time, 2) straightlining (i.e., responding with the same answer to all items on a survey), 3) inaccurate responses to a validity check question. If fraud is suspected, the study team may request additional verification of identity such as a photo ID prior to trial enrollment.

Study staff will also conduct a brief “teach back” to verify that participants understand what the study is about, potential risks, and potential benefits. Staff will document that a participant is able to describe the study. They will verify that the participant is able and willing to complete study procedures and both types of interventions, and that the participant did not have a score on the CSSRS at baseline that would make them ineligible (i.e., a positive response to CSSR-S items 3, 4, 5, and 6 [Past 3 months]). Study staff will then randomize the participant. Randomization occurs in REDCap via the press of a button. Study staff will inform the participant of the arm to which they were randomized, and then assist them with starting that intervention by connecting them to a therapist or helping them to access study yoga classes.

Participants will be considered “ENROLLED IN THE CLINICAL TRIAL” when they are randomized.

This means that some participants will consent to study participation but will not be enrolled in the clinical trial. We will track all engagement with the study, including numbers consented, numbers enrolled in the clinical trial (randomized), and reasons for lack of enrollment (e.g., not meeting inclusion criteria; lost to follow-up). No children will be enrolled.

People who speak Spanish will follow the same process as above. All materials will be translated into Spanish. Spanish-speaking staff will interact with the participants who prefer to speak Spanish.

We will not use surrogate decision makers.

It will be rare that we will enroll patients of study staff given the challenges with enrolling participants already in psychotherapy (i.e., they would need to be willing to reduce session and try a new treatment with a new provider), but if we do then we will ensure that a clinician who knows the participant, but not their treating clinician, will discuss the study with them as well as complete the informed consent procedures.

# STUDY PROCEDURES

## Study Assessments

Primary assessments occur at Baseline (week 0, prior to randomization), and weeks 6, 12, 18, and 24. Participants will also complete a very brief assessment of amount of yoga practice at weeks 3 and 9. All self-report assessments will be available in English or Spanish. When an assessment is due, participants will be sent an automated link to complete the assessments via REDCap (3 days before the due date an on the due date). In addition, they will receive reminders if they do not complete the assessment (3, 6, and 9 days after the due date), and, if needed, follow-up phone calls from study staff (starting 1 week after the due date; this will occur for weeks 6, 12, 18, and 24 assessments only). Assessments will be considered completed on time if they are done within 2 weeks of due date. It is expected that the assessments at Weeks 0 (baseline) and 24 (end of treatment) will take approximately 45 min to complete, at Week 12 will take approximately 30 min to complete and at Weeks 6 and 18 will take approximately 20 min to complete. Please see Table 1 for a description of self- report assessments.

All assessments are intended to be completed in REDCap by the participant. However, if the participant does not complete the assessments, and receives a follow-up call from study staff, and wants to complete the assessment via interview at that time, a blinded research staff member will administer the assessment as an interview.

## Description of Specific Measures

**Patient Health Questionnaire-9 (PHQ-9)** is a 9-item self-report questionnaire that is widely used to assess depression symptom severity^124-128^. A PHQ-9 score ≥10 has a sensitivity of 88% and a specificity of 88% for major depression. PHQ-9 scores of 5, 10, 15, and 20 represented mild, moderate, moderately severe, and severe depression, respectively ^124,125^.

**The World Health Organization-5 Well-Being Index^22,32^ (WHO-5)** is a 5-item self-report questionnaire with positively-worded statements related to positive mood, vitality, and general interest over the prior two weeks. The WHO-5 is reliable, has shown strong construct validity as a measure of general quality of life, and is sensitive to change[^52^](#_bookmark42)[^,53^.](#_bookmark43)

**PROMIS-29**[**^54^**](#_bookmark44) has seven health domains (physical function, fatigue, pain interference, depressive symptoms, anxiety, social functioning, sleep disturbance) using four items per domain. It has demonstrated good psychometric properties[^54^.](#_bookmark44)

**PROMIS-Anger**[^55^](#_bookmark45)[^,56^](#_bookmark46) is a 5-item self-report scale that we will use this to assess the exploratory outcome of irritability.

**Treatment History and Healthcare Utilization** is a brief measure of the quantity and characteristics of services (e.g., other psychotherapies) used by study participants. It assesses the number of days spent in inpatient and day hospital treatment psychiatric programs. This is an outcome of interest to our insurance stakeholders and is a marker of more severe illness.

**Columbia Suicide Severity Rating Scale, Screener/ Recent – Self-Report (CSSRS-SR)**[^57^](#_bookmark47) will be administered to assess suicide-related thoughts and behaviors. (See below for details regarding management of suicide risk).

**Modified Systematic Assessment for Treatment Emergent Effects (SAFTEE)**[**^58^**](#_bookmark48) is widely used in both clinical and research contexts to assess new adverse events over the course of treatment. We will use a modified, self-report version (tailored for the study interventions) that we have successfully used to track adverse events in previous studies151,152.

**Patient Preference** will be assessed by describing both treatments and then asking participants to determine which treatment is their stated preference.

**Credibility Expectance Questionnaire (CEQ)**[**^59^**](#_bookmark49) is quick and easy-to-administer scale for measuring treatment expectancy and rationale credibility. It has demonstrated high internal consistency and good test–retest reliability156.

**Multidimensional Assessment of Interoceptive Awareness-2 (MAIA-2)**[**^60^**](#_bookmark50) assesses the degree to which one experiences one’s body as safe and trustworthy. The MAIA-2 has demonstrated good psychometric properties[^60^.](#_bookmark50) We will use only the trusting scale (i.e., 4-items) based on previous research[^61-63^](#_bookmark51) and to minimize participant burden.

**Demographic and clinical survey.** We use the demographic survey to characterize the sample and assess relevant variables for this study (e.g., body weight, previous experience with study interventions, chronic medical health conditions).

**Composite International Diagnostic Interview Screening Scales (CIDI-SC)**[**^64^**](#_bookmark52) are diagnostic assessments for core disorders in the full CIDI 5.0. We will be using the Mania/Hypomania and Major Depression sections to determine a DSM-5 diagnosis of major depression and bipolar disorder over the past year for HTE analyses.

**Tobacco, Alcohol, Prescription medication, and illicit** **Substance use screening Tool Adapted (TAPS-P1)^71^** will be used this measure to assess for substance abuse.

**The Yoga Practice Questionnaire159** was developed by Dr. Uebelacker and used in her previous yoga studies to assess amount of yoga practice and class attendance to external yoga classes. It will be administered at all study visits (weeks 0, 6, 12, 18, 24).

**BA Practice Questionnaire** will be completed the BA therapist to assess participant adherence to weekly practice of BA (e.g., number of activities scheduled).

**Final WEEK 12 Open Ended Questions.** We will use this questionnaire to better understand difficulties participants face that impede their attendance to the interventions (BA sessions or yoga classes).

## Other Data Collected

Study staff (i.e., BA therapist or clinical research coordinator) will track yoga class and BA therapy session attendance weekly (i.e., did the participant attend the session and if so, for how long). These data will be recorded in REDCap by the study staff. If a participant does not attend a session, study staff will follow-up with the participant to encourage attendance to the next session as well as determine the reason for missing the visit and to problem solve potential barriers.

Table 1. Self-Report Assessments Completed in REDCap

| **Purpose of Assessment** | **Description** | **Specific measure to**  **be used** | **Timepoints**  **(weeks)** |
| --- | --- | --- | --- |
| Primary Outcome | Depressive symptoms | PHQ-9 | 0, 6, 12, 18, 24 |
| Secondary Outcome | Overall well-being | WHO-5 | 0, 6, 12, 18, 24 |
| Secondary Outcome | Anxiety | PROMIS-29 | 0, 6, 12, 18, 24 |
| Secondary Outcome | Sleep | PROMIS-29 | 0, 6, 12, 18, 24 |
| Exploratory Outcome | Physical functioning | PROMIS-29 | 0, 6, 12, 18, 24 |
| Exploratory Outcome | Social functioning | PROMIS-29 | 0, 6, 12, 18, 24 |
| Exploratory Outcome | Pain Interference | PROMIS-29 | 0, 6, 12, 18, 24 |
| Exploratory Outcome | Irritability | PROMIS-Anger | 0, 6, 12, 18, 24 |
| Exploratory Outcome | # days of psychiatric inpatient and day hospital treatment | Treatment History & Healthcare Utilization | 0, 12, 24 |
| Safety | Suicide thoughts or behaviors | CSSRS-SR | 0, 6, 12, 18, 24 |
| Safety | Adverse events | SAFTEE | 6, 12, 18, 24 |
| Heterogeneity of Treatment Effects | Patient preference | Single item | 0 |
| Heterogeneity of Treatment Effects | Credibility of intervention | CEQ | 0 |
| Heterogeneity of Treatment Effects | Expectancy of success of  intervention | CEQ | 0 |
| Heterogeneity of Treatment Effects | Experience of one’s body as  safe and trustworthy | MAIA-2 | 0 |
| Heterogeneity of Treatment Effects | Demographic and clinical  survey | Demographic survey,  including BMI | 0 |
| Heterogeneity of Treatment Effects | Diagnosis of Mood Disorder | CIDI-SC | 0 |
| Substance Use | Assessment of substance use | TAPS-P1 | 0 |
| Treatment Utilization | Other psychotherapy | Treatment History & Healthcare Utilization | 0, 12, 24 |
| Treatment Utilization | Yoga and BA Practice | Yoga and BA Practice  Questionnaires | 0, 3, 6, 9, 12, 18, 24 |
| Understanding Participant Experience | Participant’s use of skills from study interventions in everyday life | Questionnaire with open-ended questions | 12, 24 |
| Understanding Participant Experience | Participant’s difficulties which impeded their attendance to the intervention | Open ended questionnaire | 12 |

## Description of Study Interventions

**Hatha Yoga**: We will use the yoga instructor manual that Dr. Uebelacker has previously used in a trial of yoga for depression70. Yoga classes will be gentle and physically accessible for people who are naïve to yoga. Teachers will frequently guide participants to focus on their breathing and coordinate movements with breath. Teachers will offer variations on the postures and encourage participants to choose variations that provide some challenge but do not cause strain or pain. Classes will be 1 hour long and include a brief sitting meditation, warm-ups, standing postures, and a final resting meditation. All participants will be invited to attend a synchronous yoga class via a HIPAA-compliant videoconference option once per week during the first 3 months of the study (Note that, because yoga classes in Spanish will be offered only a few times per week, we will offer asynchronous (pre-recorded) option if a mono-lingual Spanish speaker has to miss the Spanish class on a particular week.) Classes will include 5-10 people, will be offered at multiple times throughout the week, and will be led by yoga teachers who have completed a Yoga-Alliance approved training program and have received further, study-specific training. We will also provide to participants a list of vetted yoga classes that are freely available online, as well as guidelines for choosing online or community yoga classes that are appropriate for them. This information should support their continued yoga practice after their initial 3 months of study participation. All classes will be audio or video record on an institutional device. Participants will always be reminded that the class will be recorded at the beginning of the session. Unless the participant specifically declines, the study staff will mail the required equipment (i.e., yoga mat).

**Behavioral Activation (BA)**: The goal of Behavioral Activation Psychotherapy is to help people identify and (re)engage in meaningful and positive activities through psychoeducation, identification of values and associated activities, goal setting, problem-solving, and monitoring of goal completion.

Engagement in meaningful and positive activities is thought to be the key mechanism of action for BA100. There will be a BA manual for therapists and training provided by study staff on the manual (e.g., asynchronous and synchronous training sessions). BA therapists will be community clinicians who are licensed mental health professionals trained to implement the content of the BA manual. BA will be provided individually via telehealth (HIPAA-compliant video or audio only) and will be billed to participant insurance. At the start of BA treatment, participants will be oriented to the time-limited nature of BA, with the expectation that they will attend 8 sessions over the course of 3 months. All participants will be invited to use worksheets provided by their therapists to support BA goals and exercises outside of sessions; this is often part of real-world clinical care. Similarly, consistent with psychotherapy guidelines and usual care, we will encourage participants to pause BA, or all psychotherapy, during the 3-month, follow-up period to practice the skills taught during the treatment phase as well as to support ongoing self-management. Importantly, this pausing of treatment will be discussed during the informed consent process as well as at the start of the BA treatment. Participants will be expected to practice their BA skills by reviewing the BA manual again on their own and continuing to use their worksheets and support from videos that we will create and post on YouTube. However, if there is a clinical need for therapy to continue during the follow-up phase, we will permit this to happen as it is consistent with real-world practice and strictly prohibiting this practice could pose a safety concern. We will track healthcare utilization throughout the study (e.g., therapy sessions during the follow-up period) to explore whether this could impact the study outcomes. All classes will be audio or video record on an institutional device. Participants will always be reminded that the class will be recorded at the beginning of the session. Unless the participant specifically declines, the study staff will mail the required equipment (i.e., notebook for use in psychotherapy sessions).

## Remuneration

Participants will be paid for completing study assessments (amount is higher for longer assessments); they will not be compensated for attending study intervention sessions. Please see Table 2 for a description of the remuneration schedule.

## Table 2. Remuneration Schedule

| **Assessment Timepoint** | **Description** | **Compensation for Completion of**  **Assessment** |
| --- | --- | --- |
| Baseline (week 0) | Baseline, pre-randomization | $40 |
| Week 6 | Brief, mid-treatment assessment | $20 |
| Week 12 | End of acute treatment | $40 |
| Week 18 | Brief, mid-follow up assessment | $20 |
| Week 24 | End of follow-up, primary outcome | $50 |
| **TOTAL** |  | **$170** |

**Communication with Study Staff**

All procedures will occur remotely. Study staff will communicate with participants via email, text, or phone call, according to participant preference. Texts messaging will occur using devices and procedures approved by the local recruitment site (all healthcare institutions). These will be institutionally owned, password-protected mobile device text messaging. If participants do not have access to a reliable device or reliable wifi, the study may loan them a phone or small tablet for use during the study. These devices will have unlimited data. Study participants will not incur any expenses for use of these devices. Devices will be owned by an institution that is part of this study, and will follow standard requirements for loaning out devices to study participants at the local institution (e.g., resetting  the device, changing the phone number between users). Emails and texts will not be encrypted, but participants will be educated about possible risks with receiving unencrypted email (see the consent form). We will not send protected health information via email or text. We will make sure participants are aware that an urgent or private information should be communicated to study staff via a telephone call. Please see more information in “Minimizing risks due to loss of confidentiality” below. Twilio may be used for sending links to redcap surveys.

**SPECIFIC TEXT TEMPLATES**:

Appointment reminders:

- **1 week prior to the assessment** (This gives the participant an opportunity to reschedule if their schedule has changed.)
  - *REMINDER: You have a COMPARE study appointment next week, on [date] at [time]. Reply “CONFIRM” to confirm now. Call this number if you would like to speak with [RA].*
- **1 day prior to the assessment**
  - *REMINDER: You have a COMPARE study appointment tomorrow, [date] at [time]. Call this number if you would like to speak with [RA] about this appointment.*
- **On the morning of the assessment**
  - REMINDER: You have a *COMPARE study* appointment TODAY, [date] at [time]. Call this number if you would like to speak with [RA] about this appointment.
- **1 day prior to a yoga class**
  - *REMINDER: You have your yoga class tomorrow, [date] at [time]. Please make sure to sign into class at least 10 minutes early, have your mat and chair with you, and position your camera so that you’re fully visible to the teacher. Reply “CONFIRM” to confirm now, or call this number if you would like to speak with [RA].*

*To join class, simply click this link: [zoom link].*

**General info re. texting**:

Administrative reasons for sending text messages to participants include:

- Scheduling and providing reminders for assessments, classes, and other research activities
- Sending REDCap links
- Sending Zoom or other video conference links

***Responding to Text Messages:***

1. Never respond to a text message from a participant that includes PHI. **Examples of messages to send back:**
   1. Please call me at [study cell #] so we may discuss more.
   2. I am not able to give you specific information in a text message. Please call me at [study cell #].
   3. The information I have for you is confidential. I can tell you more if you call. Please call me at [study cell #].
2. If a participant sends a text message indicating they are in crisis, call the participant immediately and notify the site PI. Any suicidal ideation, homicidal ideation, or indication that the participant is planning to or has hurt themselves or another individual should be reported to the site PI and, if applicable, participant’s clinical team, IRB, or emergency services. Use the standard operating procedure for further guidance.
3. Do not discuss sensitive or personal matters via text messaging.
4. If you are unsure of how to respond to a text message, consult with the site PI or co-I for a second opinion.
5. Responses to text messages should typically only occur on business days (M-F) between 8-6. Be considerate not to text participants too early in the morning, unless they have a visit scheduled and need an appointment reminder.

***Documentation:***

1. Text message communication will be logged in the tracking database in REDCap.

## Study Termination Criteria

Study participants may engage in any other community depression treatment, if needed, during the course of the study. If a participant starts a new course of psychotherapy with a non-study provider, they will not be terminated from the study. Moreover, there are circumstances that may lead to participants stopping the study interventions (e.g., hospitalization or higher level of care or a medical event, such as pregnancy, in the yoga group), but they will remain in the study and continue to complete the assessments, if possible. Therefore, there are few circumstances in which a participant may be terminated from the study. These include:

- Participant requests to end study participation.
- The study is suspended or terminated.
- The PIs withdraw a participant from the study (Such a withdrawal could occur if, in the judgment of the PIs or site PIs, participating in the study is negatively impacting the participant or someone else.)

**Procedures for Discontinuation**: Participants who discontinue from the study will continue to be followed by their treating clinician or referred to other providers and services as clinically indicated. We will also record the reason(s) for study discontinuation in the REDCap database.

## Return of Results

As part of enrollment process, we will include a question that will allow the participants to consent/reject the option of being contacted after the completion of the study to share the study results. At the end of the study, we will provide all participants that have consented to this process with a result summary that will be written in culturally competent, lay language. We will provide this summary to PCORI. This result summary will only include aggregate information without any specific participant details. We will also post the result summary on the study site website for wider distribution of the information as well as clinicaltrials.gov. Finally, we will offer local or web-based presentations at the end of the study for interested participants.

# Risks and Discomforts

## Potential Risks

***Discomfort, distress, or mild Injury***. During the study assessments, participants may experience some discomfort or anxiety from completing self-report questionnaires given that we are collecting sensitive information about the participant.

Like other forms of mild physical activity, participation in hatha yoga confers a risk of mild physical injury, including muscle aches or strains which will be clearly discussed during the consent process. We do not anticipate any serious risks to subjects due to study participation.

Participants in either intervention could experience an increase in depression or elevated symptoms or suicidal ideation related to the natural waxing and waning of symptoms. There is also a risk that not all subjects will improve with treatment; however, both study interventions are evidence-based treatments for depression, and procedures will be in place to monitor and respond to this potential risk.

***Breach of confidentiality:*** Because virtual yoga classes are group classes, there is always a risk that one participant could take actions that compromise the confidentiality of another participant (e.g., by allowing another household member to view the class with them or disclosing the name of another participant who is in the study).

There is always the possibility of a breach of confidentiality given that computer hackers have been able to breach even the most secure data storage systems. Although we consider it unlikely, it is possible when transmitting data on the Internet and thus, we will take precautions to ensure that this potential risk is minimized. We will also educate participants about this risk during the informed consent process.

With regard to study-related text or email messages, there is always a risk the message could be intercepted or sent to the wrong place. If a participant shares their cell phone or computer with others, they risk them seeing any email or text message about the study.

***Insurance copayments:*** If participants are assigned to receive BA psychotherapy, they may be responsible for insurance copayments. [The study sites may pay for costs of psychotherapy for people who are determined to be uninsured or underinsured; this determination will be made prior to randomization in collaboration with the participant.]

## Protections Against Potential Risks

***Increased distress due to assessment or intervention procedures.*** The risks of possible distress due to the assessment and treatment procedures will be minimized by: a) using assessments and procedures which have been widely used in previous research studies; b) training yoga instructors in how to minimize and manage distress that may occur in class; and c) having study investigators (who are clinical psychologists) available to talk with participants, if needed, should they report experiencing study-related distress to any member of the study research staff.

***Injuries due to the yoga or BA intervention***. We do not anticipate any serious injuries due to study participation; however, we will carefully monitor adverse events. Specifically, the SAFTEE will be administered to monitor adverse events in both groups over the study duration. We will also specifically inquire about psychiatric SAEs, such as a psychiatric hospitalization. Study staff will contact the participant to better understand the nature of the event and potential relationship to study participation for every SAE. AEs and SAEs will be reviewed by the DSMB every 6 months to enhance detection of any patterns that may develop over time.

For the yoga intervention, we will have specific procedures in place to minimize the risk of physical injury. We will: a) exclude participants with contraindicated medical conditions (as described in inclusion/ exclusion criteria); b) require all instructors to have attended at least a 200-hour Yoga Alliance teacher training program, as this ensures that they have had a training program accredited by the Yoga Alliance with attention to safety measures; and c) provide gentle yoga classes that avoid use of any complex postures that increase risk for injury. Class content will be designed to accommodate the needs of yoga-naïve students who are not currently physically active. By presenting modifications of all postures, and by using props (e.g. common household items such as a chair), the risk for injury will be minimal. We will not use postures in this study that are most commonly associated with adverse events (e.g., headstands, shoulder stands, handstands). Finally, to be cautious, if a participant becomes pregnant, we will recommend that they drop out of the study yoga classes, and consider seeking yoga classes specifically modified for pregnant women in their community as pregnant women may be at increased risk of injury due to ligament laxity, pelvic joint instability or other physical changes during pregnancy.

We do not expect any risks associated with the BA intervention other than the typical risks associated with psychotherapy, or discussing potentially sensitive information. To minimize these risks, study therapists will be trained health professions embedded in mental health centers. The study therapists will also be trained on the BA manual by study staff and provided ongoing supervision throughout the study duration to provide the most appropriate support to participants.

***Monitoring suicidal ideation (SI) and deterioration.***

Possible deterioration and suicidal ideation (SI) will be monitored every 6 weeks with the PHQ-9 and CSSRS. If a participant’s score indicates severe depression (PHQ-9 > 20) in the past 2 weeks or suicidal ideation or behavior in the past 6 weeks, we will follow our standard operating procedure (SOP), which is as follows. The participant will receive an automated message in REDCap: with: a) information about the National Suicide Prevention Lifeline (988) and website, including the fact they are a 24/7 hotline and can help if a person is at immediate risk for self-arm; b) encouragement to reach out to their local healthcare provider as needed; c) a phone number for study staff who can put them in touch with a licensed study clinician during business hours

If a participant specifically endorses thinking about a method for suicide, intention to act on their thoughts, preparatory behaviors, or an attempt within the past 6 weeks, participants will see an additional message that a clinician will outreach to that person on the next business day. REDCap will then send an alert to study clinicians (as well as site research staff) about that participant. This will prompt a licensed study clinician (including a site PI, MPIs, safety officer, or other licensed mental health professional) to outreach via phone to the participant within the next business day. This clinician will conduct a suicide risk assessment, and based on that assessment, offer support and resources for intervention. The assessment, disposition, and plan will be documented in the study record. Clinicians may also elect to follow-up again with the participant if indicated. Clinicians/study staff may also be reaching out to the participant’s emergency contact person in case they believe participants may cause harm to themselves or to others. Participants will not be discontinued from the study unless they choose to be.

***Minimizing risk due to loss of confidentiality.*** The following methods will be used to protect

participant confidentiality:

- Study data will be stored in REDCap. REDCap (Research Electronic Data Capture) is a free, secure, HIPAA compliant web-based application hosted by the Mass General Brigham Research Computing, Enterprise Research Infrastructure & Services (ERIS) group. REDCap offers multiple levels of security features ensuring access control, audit control, data integrity, and user authentication. Access to the REDCap database will require user accounts with strong passwords and only approved personnel will hold such an account.
- Study data will be reviewed only by pre-designated or IRB-approved study personnel.
- All personnel will be trained in research confidentiality procedures and will be educated

about the importance of strictly protecting participants’ rights to confidentiality.

- Synchronous yoga classes will be conducted using a HIPAA-compliant platform (e.g., Zoom). Classes will be monitored to ensure that people who should not be a part of the class do not enter electronically. We will ask participants to find as private a space as possible for their real-time yoga classes, and remind participants of this at the start of each class. All participants will be asked to keep the names of other participants in their class confidential. We will tell participants not to record classes; any recording is grounds for dismissal from the study. Finally, we will make sure all participants are aware of the possibility of a breach of privacy if another participant discloses their identity outside of the context of class. (This breach of privacy is possible in in-person classes as well).
- BA therapists will use the HIPAA-compliant portal that they typically use when providing psychotherapy services.
- Study email and text messages will be sent from study cell phones that are password protected and provided by the local site, and/or institutional email accounts (similarly provided by the local recruitment site). We will educate participants about risks of text messages and emails. We will advise them on how to protect their privacy (e.g., having password protection on a phone and/or using a private email.) We will ask for their preference re: email vs. text message. We will not send any protected health information via email or text. Rather, emails and text messages are used for scheduling, reminding participants about study sessions, and sending links to HIPAA-compliant portals for study interventions or REDCap for study assessments.

# Benefits

Potential benefits to participants include access to evidence-based treatments for depression and increased assessment and monitoring of their mood symptoms. Benefits of the study to the field include the attainment of much-needed knowledge about the effectiveness of hatha yoga in comparison to an established psychological treatment for depression (BA), as well as understanding of heterogeneity of treatment effects that can guide matching patients to treatments. We believe that most serious risks (e.g., loss of confidentiality, major psychological distress) to subjects are very unlikely. We have attempted to minimize these risks (described above). While some risks may be more likely to occur (e.g., minor, transient psychological distress; muscle soreness), these risks are not more serious than seeking other treatments for depression in the community or doing the regular physical activity that is recommended for adults. Thus, on balance, the risks to subjects are reasonable in relation to the anticipated benefits to research participants and others.

# Statistical Analysis

The primary goal of this study is to compare the effectiveness of yoga and BA for adults with clinically significant depressive symptoms (Aim 1) and identify patient-level characteristics that predict HTE (Aim 2) over 6 months. We will use intention-to-treat analyses and report results per guidelines in the CONSORT extension for non-inferiority trials177.

**Aim 1 analysis:** The primary endpoint for Aim 1 is depressive symptoms as measured by the PHQ-9. The PHQ-9 will be administered at baseline (i.e., week 0, prior to randomization) and every 6 weeks during the 6-month follow up (i.e., weeks 6, 12, 18, 24). First, we will carry out an exploratory data analysis by calculating descriptive statistics (e.g., mean, SD, min/max) and generating data visualizations (e.g., box plots, histograms) of the outcome at each assessment visit by intervention group, allowing us to identify potential outliers and anticipate reasonable regression modeling assumptions. Then, we will fit a linear mixed effects model with random participant-level intercepts and slopes and fixed effects for intervention, time, an intervention-by-time interaction, and a fixed categorical effect for site (given that randomization is stratified by site). Non-linear trends over time will be modeled using smoothing splines. Mixed models incorporate information from all available longitudinal data and account for within- participant correlation due to repeated measures. Based on this model, we will estimate the between- group difference in mean PHQ-9 scores at 6 months and calculate a two-sided 95% confidence interval (CI) of this difference to assess the hypothesis that yoga is non-inferior to BA for depressive symptoms (using a margin of 2 points) over 6-months.

Secondary endpoints for Aim 1 include well-being, anxiety, and sleep. After exploratory data analysis, linear mixed effects models similar to those used for the primary endpoint will be fit to assess whether yoga is non-inferior to BA for each of these secondary outcomes. Model-based estimates and 95% CIs of between-group differences in mean outcome scores at 6 months will be compared to prespecified non-inferiority margins for each outcome (see **Table 3**).

Sensitivity analyses will be conducted for primary and secondary endpoints by further adjusting regression models (and, thus, model-based estimates and CIs) for any baseline imbalances between intervention groups. In addition to our primary intent-to-treat analyses, we will also carry out per- protocol analyses (e.g., using causal inference g-methods178) to assess whether differential treatment engagement/adherence or intervention modality during the study impact the comparative effectiveness of yoga and BA.

**Aim 2 analysis:** Although in Aim 1 we hypothesized non-inferiority of yoga to BA in aggregate (i.e., across all study participants), we anticipate that for certain individuals, yoga will be superior to BA, and for others, BA will be superior to yoga. The goal of Aim 2, therefore, is to determine whether certain pre-specified participant-level characteristics at baseline predict HTE for the primary endpoint of PHQ-9 depressive symptoms at 6 months. Prespecified moderators, or HTE variables, include 1) preference for yoga or BA, 2) perceived credibility of yoga or BA, 3) depression severity, and 4) body trust. Specifically, we expect greater effectiveness of yoga compared to BA among participants with a preference for yoga, higher perceived credibility of yoga relative to BA, less severe depression, and more body trust at baseline. Correspondingly, we expect greater effectiveness of BA compared to hatha yoga among participants with a preference for BA, higher perceived credibility of BA relative to yoga, more severe depression, and less body trust at baseline. We will examine whether each anticipated moderator predicts HTE for depressive symptoms by adding interaction terms, as appropriate, to the PHQ-9 linear

mixed effects models described above. Specifically, for each moderator, we will fit an expanded mixed model by adding fixed effects for the moderator, two-way moderator-by-intervention and moderator- by-time interactions, and a three-way moderator-by-intervention-by-time interaction: this three-way interaction term represents the estimated differential intervention effect across levels of the moderator. HTE will be formally assessed using a likelihood ratio test by comparing this expanded model to one without the three-way interaction term. To account for multiple testing in Aim 2 of these four anticipated moderators, a Bonferroni corrected p-value threshold of 0.05/4 = 0.0125 will be used for each likelihood ratio test. Model-based HTE estimates and 95% CIs will be calculated and presented in an interpretable fashion using tables and figures.

**Exploratory analyses:** *Exploratory outcomes:* We will compare the effectiveness of yoga vs. BA over 6 months for additional exploratory outcomes (see **Table 1**). We will conduct exploratory data analyses and regression modeling as described in Aim 1. For non-continuous outcome types (e.g. count, binary), we will use generalized linear mixed effects models. We will not formally assess non-inferiority for these exploratory outcomes but instead report model-based estimates and CIs for comparing the intervention groups (without reference to a particular margin).

*Exploratory Moderator or HTE analyses:* Given mixed empirical results and lack of a theoretical rationale, demographic variables (i.e., race, ethnicity, age, sex), diagnosis (i.e., bipolar vs unipolar depression), and other treatment (e.g., pharmacotherapy for depression) will be assessed as potential moderators in exploratory HTE analyses. We will use the same modeling strategy (i.e., addition of interaction terms and likelihood ratio tests) as described in Aim 2 and will report unadjusted p-values and 95% CIs.

**Loss to follow-up and missing data:** The study team will do everything possible to minimize missing data and loss to follow-up. We will provide thorough summaries of reasons for missingness, proportions of missing data, and test for differences in the characteristics of patients with and without missing data, and we will describe these and the implications of missing data for trial interpretation when reporting results. Our primary linear mixed effects models in Aims 1 and 2 incorporate all available data both from participants who complete the study and those who dropout prior to 6 months. Additionally, we will use multiple imputation to account for observed auxiliary individual-level covariates that are expected to predict missed participant visits and dropout. Sensitivity analyses will be carried out to assess the potential impact of unobserved predictors of dropout on trial results[^65-67^.](#_bookmark53)

## Sample size and power

We will randomize 518 adults with clinically significant depressive symptoms across 2 treatment arms (i.e., 259/arm). For the primary PHQ-9 endpoint at 6 months, assuming a SD of 7 in both arms[^68^](#_bookmark54)[^,69^,](#_bookmark55) an actual mean difference of zero between arms, a non-inferiority margin of 2 points (see previous studies used to make this choice[^40^](#_bookmark33)[^,70^](#_bookmark56)), and 25% dropout prior to the 6-month visit (based on previous research with synchronous behavioral interventions[^12^](#_bookmark11)[^,34^](#_bookmark30)), this sample size will provide 80% power to detect non- inferiority of hatha yoga to BA based on a one-sided two-sample equal-variance t-test with a significance level of 0.025. We based our calculation on a conservative 0.025-level one-sided test because 1) this test corresponds with calculating the more conventional two-sided 95% CI and comparing the CI bounds to the non-inferiority margin and 2) the resulting larger sample size will provide more power to detect meaningful HTE in Aim 2 and our exploratory analyses. Additionally, this sample size will provide at least 84% power to assess non-inferiority for each of the secondary outcomes of overall well-being, anxiety, and sleep over the 6-month study period. All power calculations were based on SD estimates from prior studies and non-inferiority margins below what is considered clinically meaningful (see **Table 3**).

## Table 3. Non-inferiority margins and power estimates.

| **Outcome** | **Description** | **Specific measure to be**  **used** | **Non- inferiority**  **margin** | **Anticipated SD** | **Power estimate^** |
| --- | --- | --- | --- | --- | --- |
| Primary | Depressive symptoms | PHQ-9^a^ | 2 points | 7 points | 80% |
| Secondary | Overall well-being | WHO-5^b^ | 1.5 points | 5 points | 84% |
| Secondary | Anxiety | PROMIS-29^c^ | 3 points | 10 points | 84% |
| Secondary | Sleep | PROMIS-29^c^ | 3 points | 10 points | 84% |

a Range of possible scores = 0-27. b Range of possible scores = 0-25. c Converted to T-scores, i.e., with a mean = 50 and SD = 10.

^ These estimates are based on other previous studies137,138-142 as well as our clinical trials: NCT03844321 and NCT03373110.

# Monitoring and Quality Assurance

## Adverse Event Criteria and Reporting Procedures

The MPIs (Drs. Sylvia and Uebelacker) will have responsibility for monitoring the integrity of study data and participant safety. Dr. Weinstock, Co-Investigator and the Chief Safety Officer, will assist with this process given her expertise.

Adverse events will be identified via: a) participant’s report on the SAFTEE; or b) participant’s report of

an AE to a study staff member or study interventionist.

The research team will meet weekly to review the progress of participants in the study. We will discuss and resolve any safety issues more frequently if necessary as well as any concerns from the treating clinicians, study staff, DSMB, IRB, or PCORI. This review will include a discussion of any SAEs that have occurred, as well as any AEs possibly, probably, or definitely related to study participation, a review and reassessment of possible risks to participants, DSMB reports and any ethical issues that may arise logs.

## Reporting Adverse Events.

- - 1. Any SAEs (e.g., death, suicide attempt, inpatient hospitalization) that are considered possibly, probably or definitely related to study participation, according to the Office for Human Research Protections (OHRP) and FDA will be reported within 36 hours to the MGH IRB. A full written report will be sent within 1 week to both the MGH IRB and to PCORI.
    2. Serious unanticipated problems (that are not SAEs) will be reported to the IRB and PCORI within 5-7 days. Any other unanticipated problems will be reported to the IRB within 2 weeks in accordance with the guidelines of OHRP and FDA
    3. All adverse events will be summarized in the PCORI and IRB annual progress reports.
    4. Study MPIs will notify PCORI promptly, but no later than 10 days after reporting any serious unanticipated problems relating to the research study to the sponsor, DSMB, IRB, the FDA, or other regulatory or oversight body; and any decision, finding, recommendation, action or direction of a DSMB, IRB, the FDA, or any other regulatory or oversight body relating to any serious unanticipated problems (e.g., serious adverse event, serious safety issue, or other serious problem.
    5. The DSMB will review a summary table of a) all SAEs; and b) any AEs possibly, probably, or definitely related to study participation twice per year.

**Additional Safety Monitoring**. The site PIs, MPIs and other study staff will discuss participant safety of the study in person or via weekly conference call. They will also discuss and resolve any safety issues more frequently, if necessary. Site PIs, in consultation with MPIs, will prepare a written report that summarizes these discussions and any decisions that are made pertaining to participant disposition. A

modified version of the SAFTEE will be administered to monitor adverse events. We will also specifically inquire about psychiatric SAEs (e.g., psychiatric hospitalization).

## Data and Safety Monitoring Board

**Functions of the External Data and Safety Monitoring Board (DSMB)**. This group will be responsible for ongoing monitoring of the trial. The MPIs, along with Dr. Weinstock, will ensure that the protocol and Data and Safety Monitoring Plan is reviewed and approved by the DSMB. Subsequently, the DSMB will meet via teleconference every six months. After meeting, the DSMB Chair will approve the minutes.

**Membership of the DSMB.** The DSMB is comprised of members who possess a high degree of competence and experience as well as the ability to function independently of all other parties involved in the study. The DSMB members function free of the career and financial interests of its members. The DSMB consists of four members (one who serves as Chair) with expertise in yoga, evidence-based psychotherapy for depression, and/or biostatistics, and a thorough knowledge of clinical trial ethics and human subject protection issues, especially for online studies.

**Functional Organization of the DSMB**. The Chairperson of the DSMB communicates with the other members to review SAEs possibly, probably, or definitely related to study participation will occur within a week of receiving any new SAE report. Reporting and communication about other matters will occur at each meeting, or every 6 months, for the duration of the study. Decisions of the DSMB will be made based on a majority vote of the members.

**Annual DSMB Report**. The DSMB will prepare a full report annually of its findings regarding safety and quality based on data received to that point in the study. This report will include a summary of all safety findings over the past year as well as an assessment of protocol compliance and data quality. Any recommendations to improve patient safety, protocol adherence, or data quality will be made in the annual DSMB report and sent to the IRB.

## Interim Data Analyses

No interim data analyses are planned.

## Data Quality and Monitoring Reports

Data Quality Reports

A Co-Investigator and statistical programmer will generate monthly and as-needed data quality reports to identify potential issues in the REDCap data. These issues will include, but are not limited to, checks on soft validation checks from REDCap, missing key data elements, logic checks, form completeness, and other issues found in the data as the study progresses. Site staff will be sent a copy of the data quality report appropriate for their role and required to comment on and resolve data issues through REDCap. If the query cannot be resolved, site coordinators should still close the query, but leave a comment as to why the query remains unresolved. Any queries which are closed will be removed from the “Open /

unresolved issues” queue and moved to the “Closed /resolved issues” section of future reports.

Although these data quality reports are scheduled to be done monthly, reports will be prepared weekly during the initial startup of the study. Major or consistent issues with data, as seen on the data quality reports (and other data reports), will be flagged by the statistical programmer and brought to the attention of the Data Committee (Dr. Rabideau, Chair) and the MPIs (Drs. Sylvia and Uebelacker).

Enrollment and Monitoring Reports

The statistical programmer will also generate monthly and as-needed enrollment and monitoring reports that will be distributed to and reviewed by clinical sites and the Steering Committee. These reports will include numbers of participants of screened, enrolled, randomized, and retained since study initiation, overall and by site. These reports will also include descriptive statistics of key baseline characteristics to ensure a diverse and representative study sample. Reports will also go to PCORI on a monthly basis.

## Study Stopping Rules

The study will be stopped if the MPIs, DSMB, PCORI, the IRB or any other regulatory oversight recommends that the study stop for any reason.

# Privacy and Confidentiality

☒ Study procedures will be conducted in a private setting. Participants will complete study procedures in their own homes, and we advise them on steps they can take to maintain privacy of their data and information about study participation.

☒ Only data and/or specimens necessary for the conduct of the study will be collected

☒ Data collected (paper and/or electronic) will be maintained in a secure location with appropriate protections such as password protection, encryption, physical security measures (locked files/areas)

- Specimens collected will be maintained in a secure location with appropriate protections (e.g. locked storage spaces, laboratory areas). Not applicable.

☒ Data and specimens will only be shared with individuals who are members of the IRB-approved research team or approved for sharing as described in this IRB protocol

☒ Data and/or specimens requiring transportation from one location or electronic space to another will be transported only in a secure manner (e.g. encrypted files, password protection, using chain-of-custody procedures, etc.)

- All electronic communication with participants will comply with Mass General Brigham secure communication policies

☒ Identifiers will be coded or removed as soon as feasible and access to files linking identifiers with coded data or specimens will be limited to the minimal necessary members of the research team required to conduct the research

☒ All staff are trained on and will follow the Mass General Brigham policies and procedures for maintaining appropriate confidentiality of research data and specimens

☒ The PI will ensure that all staff implement and follow any Research Information Service Office (RISO) requirements for this research

- Additional privacy and/or confidentiality protections

# References

1. Goldney RD, Fisher LJ, Dal Grande E, Taylor AW. Subsyndromal depression: prevalence, use of health services and quality of life in an Australian population. *Soc Psychiatry Psychiatr Epidemiol.* Apr 2004;39(4):293-298.
2. Chachamovich E, Fleck M, Laidlaw K, Power M. Impact of major depression and subsyndromal symptoms on quality of life and attitudes toward aging in an international sample of older adults. *Gerontologist.* Oct 2008;48(5):593-602.
3. Horwath E, Johnson J, Klerman GL, Weissman MM. Depressive symptoms as relative and attributable risk factors for first-onset major depression. *Arch Gen Psychiatry.* Oct 1992;49(10):817-823.
4. Cuijpers P, Smit F, Oostenbrink J, de Graaf R, Ten Have M, Beekman A. Economic costs of minor depression: a population-based study. *Acta Psychiatr Scand.* Mar 2007;115(3):229-236.
5. Cuijpers P, Vogelzangs N, Twisk J, Kleiboer A, Li J, Penninx BW. Differential mortality rates in major and subthreshold depression: meta-analysis of studies that measured both. *Br J Psychiatry.* Jan 2013;202(1):22-27.
6. APA. *Clinical Practice Guideline for the treatment of depression across three age cohorts.*

Washington DC: American Psychological Association; February 16 2019.

1. Dorow M, Lobner M, Pabst A, Stein J, Riedel-Heller SG. Preferences for Depression Treatment Including Internet-Based Interventions: Results From a Large Sample of Primary Care Patients. *Frontiers in psychiatry.* 2018;9:181.
2. Solomon D, Adams J. The use of complementary and alternative medicine in adults with depressive disorders. A critical integrative review. *J Affect Disord.* Jul 1 2015;179:101-113.
3. Newins AR, Wilson SM, Hopkins TA, Straits-Troster K, Kudler H, Calhoun PS. Barriers to the use of Veterans Affairs health care services among female veterans who served in Iraq and Afghanistan. *Psychol Serv.* Aug 2019;16(3):484-490.
4. Marks IM, Cavanagh K, Gega L. *Hands-on help: Computer-aided psychotherapy.* New York, NY, US: Psychology Press; 2007.
5. Larson-Meyer DE. A Systematic Review of the Energy Cost and Metabolic Intensity of Yoga.

*Medicine and science in sports and exercise.* Aug 2016;48(8):1558-1569.

1. Brinsley J, Schuch F, Lederman O, et al. Effects of yoga on depressive symptoms in people with mental disorders: a systematic review and meta-analysis. *Br J Sports Med.* May 18 2020.
2. Zou L, Yeung A, Li C, et al. Effects of Meditative Movements on Major Depressive Disorder: A Systematic Review and Meta-Analysis of Randomized Controlled Trials. *Journal of clinical medicine.* Aug 1 2018;7(8).
3. Hopko DR, Armento ME, Robertson SM, et al. Brief behavioral activation and problem-solving therapy for depressed breast cancer patients: randomized trial. *J Consult Clin Psychol.* Dec 2011;79(6):834-849.
4. Clarke TC, Barnes PM, Black LI, Stussman BJ, Nahin RL. Use of Yoga, Meditation, and Chiropractors Among U.S. Adults Aged 18 and Over. *NCHS data brief.* Nov 2018(325):1-8.
5. Center PR. U.S. adults regularly turn to a variety of activities to help cope with coronavirus outbreak. 2020.
6. Kabat-Zinn J. *Full catastrophe living: Using the wisdom of your body and mind to face stress, pain, and illness.* New York, NY: Random House Publishing Group; 2013.
7. Segal ZV, J.M.G. W, Teasdale JD. *Mindfulness-based cognitive therapy for depression.* 2nd ed. New York: Guilford Pess; 2013.
8. Segal ZV, Williams JMG, Teasdale JD. *Mindfulness-based cognitive therapy for depression.* New York: The Guilford Press; 2002.
9. Teasdale JD, Segal ZV, Williams JM, Ridgeway VA, Soulsby JM, Lau MA. Prevention of relapse/recurrence in major depression by major depression by mindfulness-based cognitive therapy. *J Consult Clin Psychol.* 2000;68(4):615-623.
10. Huberty J, Eckert R, Larkey L, Gowin K, Mitchell J, Mesa R. Perceptions of Myeloproliferative Neoplasm Patients Participating in an Online Yoga Intervention: A Qualitative Study. *Integr Cancer Ther.* Dec 2018;17(4):1150-1162.
11. Joseph RP, Durant NH, Benitez TJ, Pekmezi DW. Internet-Based Physical Activity Interventions.

*Am J Lifestyle Med.* Jan 2014;8(1):42-68.

1. Alcorn T. Is This the End of the New York Yoga Studio? 2020. https://[www.nytimes.com/2020/09/17/nyregion/coronavirus-nyc-yoga-](http://www.nytimes.com/2020/09/17/nyregion/coronavirus-nyc-yoga-studios.html?searchResultPosition=1) [studios.html?searchResultPosition=1.](http://www.nytimes.com/2020/09/17/nyregion/coronavirus-nyc-yoga-studios.html?searchResultPosition=1)
2. Alliance YY. Professional Resources. https://yourya.org/professional-resources/.
3. Bedfordshire Uo. University celebrates success of virtual yoga classes. 2020; https://[www.beds.ac.uk/news/2020/june/university-celebrates-success-of-virtual-yoga-classes/](http://www.beds.ac.uk/news/2020/june/university-celebrates-success-of-virtual-yoga-classes/).
4. Cording J. How COVID-19 Is Transforming The Fitness Industry. 2020.
5. Wernet K. 5 Wellness Trends to Watch in 2021. 2021; https://[www.mindbodyonline.com/business/education/blog/5-wellness-trends-watch-](http://www.mindbodyonline.com/business/education/blog/5-wellness-trends-watch-2021?_gl=1%2Ar951lj%2A_ga%2ANjIwODg1ODk3LjE2MTU4MTgwNjQ.%2A_ga_EDX6GRS9K2%2AMTYxNTgxODA2NC4xLjEuMTYxNTgxODIzMC4w) [2021?_gl=1*r951lj*_ga*NjIwODg1ODk3LjE2MTU4MTgwNjQ.*_ga_EDX6GRS9K2*MTYxNTgxODA](http://www.mindbodyonline.com/business/education/blog/5-wellness-trends-watch-2021?_gl=1%2Ar951lj%2A_ga%2ANjIwODg1ODk3LjE2MTU4MTgwNjQ.%2A_ga_EDX6GRS9K2%2AMTYxNTgxODA2NC4xLjEuMTYxNTgxODIzMC4w) [2NC4xLjEuMTYxNTgxODIzMC4w](http://www.mindbodyonline.com/business/education/blog/5-wellness-trends-watch-2021?_gl=1%2Ar951lj%2A_ga%2ANjIwODg1ODk3LjE2MTU4MTgwNjQ.%2A_ga_EDX6GRS9K2%2AMTYxNTgxODA2NC4xLjEuMTYxNTgxODIzMC4w).
6. Huberty J, Sullivan M, Green J, et al. Online yoga to reduce post traumatic stress in women who have experienced stillbirth: a randomized control feasibility trial. *BMC Complement Med Ther.* Jun 5 2020;20(1):173.
7. Schulz-Heik RJ, Meyer H, Mahoney L, et al. Results from a clinical yoga program for veterans: yoga via telehealth provides comparable satisfaction and health improvements to in-person yoga. *BMC Complement Altern Med.* Apr 4 2017;17(1):198.
8. Abbott R. Treating the health care crisis: complementary and alternative medicine for PPACA.

*DePaul J. Health Care L.* 2011;14:35.

1. NICE. Depression in adults: recognition and management

Clinical guideline. 2009; https://[www.nice.org.uk/guidance/cg90/chapter/Recommendations#step-1-](http://www.nice.org.uk/guidance/cg90/chapter/Recommendations#step-1-recognition-assessment-and-initial-management) [recognition-assessment-and-initial-management](http://www.nice.org.uk/guidance/cg90/chapter/Recommendations#step-1-recognition-assessment-and-initial-management).

1. Farmer MM, McGowan M, Yuan AH, Whitehead AM, Osawe U, Taylor SL. Complementary and Integrative Health Approaches Offered in the Veterans Health Administration: Results of a National Organizational Survey. *J Altern Complement Med.* Mar 2021;27(S1):S124-s130.
2. Collaborators GDaIIaP. Global, regional, and national incidence, prevalence, and years lived with disability for 354 diseases and injuries for 195 countries and territories, 1990-2017: a systematic analysis for the Global Burden of Disease Study 2017. *Lancet.* Nov 10 2018;392(10159):1789- 1858.
3. Cuijpers P, van Straten A, Warmerdam L. Behavioral activation treatments of depression: a meta-analysis. *Clin Psychol Rev.* Apr 2007;27(3):318-326.
4. Carlbring P, Hägglund M, Luthström A, et al. Internet-based behavioral activation and acceptance-based treatment for depression: a randomized controlled trial. *J Affect Disord.* Jun 2013;148(2-3):331-337.
5. Moshier SJ, Otto MW. Behavioral activation treatment for major depression: A randomized trial of the efficacy of augmentation with cognitive control training. *J Affect Disord.* Mar 1 2017;210:265-268.
6. Cook JM, Biyanova T, Elhai J, Schnurr PP, Coyne JC. What do psychotherapists really do in practice? An Internet study of over 2,000 practitioners. *Psychotherapy (Chic).* Jun 2010;47(2):260-267.
7. Crits-Christoph P, Goldstein E, King C, et al. A Feasibility Study of Behavioral Activation for Major Depressive Disorder in a Community Mental Health Setting. *Behavior therapy.* 2021/01/01/ 2021;52(1):39-52.
8. Weobong B, Weiss HA, McDaid D, et al. Sustained effectiveness and cost-effectiveness of the Healthy Activity Programme, a brief psychological treatment for depression delivered by lay counsellors in primary care: 12-month follow-up of a randomised controlled trial. *PLoS Med.* Sep 2017;14(9):e1002385.
9. Richards DA, Rhodes S, Ekers D, et al. Cost and Outcome of BehaviouRal Activation (COBRA): a randomised controlled trial of behavioural activation versus cognitive-behavioural therapy for depression. *Health Technol Assess.* Aug 2017;21(46):1-366.
10. Egede LE, Acierno R, Knapp RG, et al. Psychotherapy for depression in older veterans via telemedicine: a randomised, open-label, non-inferiority trial. *Lancet Psychiatry.* Aug 2015;2(8):693-701.
11. Osenbach JE, O'Brien KM, Mishkind M, Smolenski DJ. Synchronous telehealth technologies in psychotherapy for depression: a meta-analysis. *Depress Anxiety.* Nov 2013;30(11):1058-1067.
12. Salisbury C, O'Cathain A, Edwards L, et al. Effectiveness of an integrated telehealth service for patients with depression: a pragmatic randomised controlled trial of a complex intervention. *Lancet Psychiatry.* Jun 2016;3(6):515-525.
13. Reay RE, Looi JC, Keightley P. Telehealth mental health services during COVID-19: summary of evidence and clinical practice. *Australas Psychiatry.* Oct 2020;28(5):514-516.
14. Castro A, Gili M, Ricci-Cabello I, et al. Effectiveness and adherence of telephone-administered psychotherapy for depression: A systematic review and meta-analysis. *J Affect Disord.* Jan 1 2020;260:514-526.
15. Au A, Yip HM, Lai S, et al. Telephone-based behavioral activation intervention for dementia family caregivers: Outcomes and mediation effect of a randomized controlled trial. *Patient Educ Couns.* Nov 2019;102(11):2049-2059.
16. Choi NG, Marti CN, Wilson NL, et al. Effect of Telehealth Treatment by Lay Counselors vs by Clinicians on Depressive Symptoms Among Older Adults Who Are Homebound: A Randomized Clinical Trial. *JAMA Netw Open.* Aug 3 2020;3(8):e2015648.
17. Wosik J, Fudim M, Cameron B, et al. Telehealth transformation: COVID-19 and the rise of virtual care. *J Am Med Inform Assoc.* Jun 1 2020;27(6):957-962.
18. Koonin LM, Hoots B, Tsang CA, et al. Trends in the Use of Telehealth During the Emergence of the COVID-19 Pandemic - United States, January-March 2020. *MMWR Morb Mortal Wkly Rep.* 2020;69(43):1595-1599.
19. Maj M, Stein DJ, Parker G, et al. The clinical characterization of the adult patient with depression aimed at personalization of management. *World Psychiatry.* Oct 2020;19(3):269-293.
20. Lejuez CW, Hopko DR, Acierno R, Daughters SB, Pagoto SL. Ten year revision of the brief behavioral activation treatment for depression: revised treatment manual. *Behav Modif.* Mar 2011;35(2):111-161.
21. Topp CW, Ostergaard SD, Sondergaard S, Bech P. The WHO-5 Well-Being Index: a systematic review of the literature. *Psychother Psychosom.* 2015;84(3):167-176.
22. Wu SF. Rapid Screening of Psychological Well-Being of Patients with Chronic Illness: Reliability and Validity Test on WHO-5 and PHQ-9 Scales. *Depress Res Treat.* 2014;2014:239490.
23. Hays RD, Spritzer KL, Schalet BD, Cella D. PROMIS(®)-29 v2.0 profile physical and mental health summary scores. *Qual Life Res.* Jul 2018;27(7):1885-1891.
24. Pilkonis PA, Choi SW, Reise SP, Stover AM, Riley WT, Cella D. Item banks for measuring emotional distress from the Patient-Reported Outcomes Measurement Information System (PROMIS®): depression, anxiety, and anger. *Assessment.* Sep 2011;18(3):263-283.
25. Pilkonis PA, Yu L, Dodds NE, Johnston KL, Maihoefer CC, Lawrence SM. Validation of the depression item bank from the Patient-Reported Outcomes Measurement Information System (PROMIS) in a three-month observational study. *J Psychiatr Res.* Sep 2014;56:112-119.
26. Posner K, Brown GK, Stanley B, et al. The Columbia-Suicide Severity Rating Scale: initial validity and internal consistency findings from three multisite studies with adolescents and adults. *Am J Psychiatry.* Dec 2011;168(12):1266-1277.
27. Levine JL, Schooler NR. SAFTEE: a technique for the systematic assessment of side effects in clinical trials. *Psychopharmacology Bulletin.* 1986;22:343-381.
28. Devilly GJ, Borkovec TD. Psychometric properties of the credibility/ expectancy questionnaire.

*Journal of Behavior Therapy and Experimental Psychiatry.* 2000;31:73-86.

1. Mehling WE, Acree M, Stewart A, Silas J, Jones A. The Multidimensional Assessment of Interoceptive Awareness, Version 2 (MAIA-2). *PloS one.* 2018;13(12):e0208034.
2. Alleva JM, Tylka TL, van Oorsouw K, et al. The effects of yoga on functionality appreciation and additional facets of positive body image. *Body Image.* 2020/09/01/ 2020;34:184-195.
3. Mahlo L, Tiggemann M. Yoga and positive body image: A test of the Embodiment Model. *Body Image.* Sep 2016;18:135-142.
4. Tihanyi BT, Böőr P, Emanuelsen L, Köteles F. 'Mediators between Yoga Practice and Psychological Well-Being: Mindfulness, Body Awareness and Satisfaction with Body Image'. *European Journal of Mental Health.* 2016;11(1-2):112.
5. Sheehan DV, Lecrubier Y, Sheehan KH, et al. The Mini-International Neuropsychiatric Interview (M.I.N.I.): the development and validation of a structured diagnostic psychiatric interview for DSM-IV and ICD-10. *J Clin Psychiatry.* 1998;59 Suppl 20:22-33;quiz 34-57.
6. Moreno-Betancur M, Chavance M. Sensitivity analysis of incomplete longitudinal data departing from the missing at random assumption: Methodology and application in a clinical trial with drop-outs. *Stat Methods Med Res.* Aug 2016;25(4):1471-1489.
7. Scharfstein DO, McDermott A. Global sensitivity analysis of clinical trials with missing patient- reported outcomes. *Stat Methods Med Res.* May 2019;28(5):1439-1456.
8. Tompsett DM, Leacy F, Moreno-Betancur M, Heron J, White IR. On the use of the not-at-random fully conditional specification (NARFCS) procedure in practice. *Stat Med.* Jul 10 2018;37(15):2338-2353.
9. Löwe B, Unützer J, Callahan CM, Perkins AJ, Kroenke K. Monitoring depression treatment outcomes with the patient health questionnaire-9. *Medical care.* 2004:1194-1201.
10. Kroenke K, Wu J, Yu Z, et al. Patient Health Questionnaire Anxiety and Depression Scale: Initial Validation in Three Clinical Trials. *Psychosom Med.* Jul-Aug 2016;78(6):716-727.
11. Saxon D, Ashley K, Bishop-Edwards L, et al. A pragmatic randomised controlled trial assessing the non-inferiority of counselling for depression versus cognitive-behaviour therapy for patients in primary care meeting a diagnosis of moderate or severe depression (PRaCTICED): Study protocol for a randomised controlled trial. *Trials.* Mar 1 2017;18(1):93.
12. McNeely, J., Wu, L.T., Subramaniam, G., Sharma, G., Cathers, L.A., et al. Performance of the Tobacco, Alcohol, Prescription Medication, and Other Substance Use (TAPS) Tool for Substance Use Screening in Primary Care Patients. Annals of Internal Medicine, in press. 2016.

**APPENDIX A**

# Data Monitoring Committee / Data and Safety Monitoring Board Appendix

- - *To be completed for studies monitored by Data Monitoring Committee (DMC) or Data and Safety Monitoring Board (DSMB) if a full DMC/DSMB charter is not available at the time of initial IRB review.*
  - *DMC/DSMB Charter and/or Roster can be submitted to the IRB later via Amendment, though these are not required.*

A Data Monitoring Committee (DMC) or Data and Safety Monitoring Board (DSMB) will be convened for safety monitoring of this research study. The following characteristics describe the DMC/DSMB convened for this study (Check all that apply):

☒ The DMC/DSMB is independent from the study team and study sponsor.

☒ A process has been implemented to ensure absence of conflicts of interest by DMC/DSMB members.

☒ The DMC/DSMB has the authority to intervene on study progress in the event of safety concerns, e.g., to suspend or terminate a study if new safety concerns have been identified or need to be investigated.

☒ Describe number and types of (i.e., qualifications of) members:

The DSMB members function free of the career and financial interests of its members. The DSMB consists of four members (one who serves as Chair) with expertise in yoga, evidence-based psychotherapy for depression, and/or biostatistics, and a thorough knowledge of clinical trial ethics and human subject protection issues, especially for online studies.

☒ Describe planned frequency of meetings:

Every 6 months; DSMB members may choose to convene in between these scheduled meetings if needed.

☒ DMC/DSMB reports with no findings (i.e., “continue without modifications”) will be submitted

to the IRB at the time of Continuing Review.

☒ DMC/DSMB reports with findings/modifications required will be submitted promptly (within 5 business days/7 calendar days of becoming aware) to the IRB as an Other Event.
